# Association of Internet addiction with psychiatric symptom levels and sleep disorders: a systematic review and meta-analysis

**DOI:** 10.3389/fpsyg.2025.1573058

**Published:** 2025-04-17

**Authors:** Yuanlin Sun, Zhen Wang, Tianzhi Liu

**Affiliations:** Institute of Psychology, Liaoning Normal University, Dalian Liaoning, China

**Keywords:** meta-analysis, Internet addiction, depression, anxiety, stress, sleep disorders

## Abstract

**Background:**

Maladaptive Internet use is defined as Internet addiction disorders (IAD), which can lead to psychological problems and sleep disorders. Although many studies on the correlation between Internet addiction, psychiatric symptom levels, and sleep disorders have been conducted in recent years, there is no meta-analysis to substantiate the connection between these variables.

**Methods:**

We systematically searched databases including Web of Science, PubMed, Embase, and the Cochrane Library to collect relevant studies using keywords associated with Internet addiction, psychological problems, and sleep disorders. All comparable studies that provided sufficient data (e.g., correlation coefficients) were included in our analysis.

**Results:**

41 studies were included, and the results indicated that IAD was associated with psychological problems and sleep quality at moderate to low levels (depression: *r* = 0.39, 95%CI = 0.34–0.45; SMD = 1.34, 95%CI = 0.81–1.86; OR = 0.86, 95%CI = 0.46–1.26; anxiety: *r* = 0.30, 95%CI = 0.23–0.37; OR = 0.90, 95%CI = 0.29–1.52; stress: *r* = 0.34, 95%CI = 0.29–0.38; OR = 1.76, 95%CI = 0.37–3.16; sleep problems: *r* = 0.26, 95%CI = 0.19–0.33).

**Conclusion:**

This meta-analysis reveals that IAD is positively associated with depression, anxiety, and sleep problems, which indicates that individuals with IAD have an increased risk of depression, anxiety, and sleep problems. Hence, high attention should be paid to Internet addictive behaviors, and preventive and treatment measures should be adopted timely.

**Systematic review registration:**

The publicly accessible registration record can be found at: https://www.crd.york.ac.uk/prospero/.

## 1 Introduction

Internet overuse has been linked to maladaptive behavior, “Internet addiction,” also known as “pathological Internet use,” “compulsive Internet use,” and “Internet overuse.” Internet addiction is defined as the behavior of using the Internet pathologically or compulsively, which disrupts the individual’s normal life (Kumar et al., 2018; Lozano-Blasco and Cortes-Pascual, 2020). Internet addiction has become a significant issue globally, particularly among adolescents and young adults, with a 20–30% prevalence rate (Azmi et al., 2019; Černja et al., 2019).

Excessive Internet use will lead to a range of physical and mental problems such as anxiety (Geng et al., 2021; Sayed et al., 2022), depression (Chi et al., 2019; Ge et al., 2023; Haand and Zhao, 2020; Yuan et al., 2021), and stress (Javaeed et al., 2019; Kayis, 2022). The link between Internet addiction and depression has been reported. For instance, previous studies have pointed out that there is a strong positive correlation between Internet addiction and depression (Liu et al., 2018; Suk-Hyun Hwang et al., 2015; Wong et al., 2020). People with symptoms of Internet addiction are more likely to experience depressive symptoms, and some people with depression may adopt maladaptive coping strategies (e.g., Internet addiction) to alleviate their symptoms. According to the Compensatory Internet Use Theory, individuals with depression and anxiety are more likely to use the Internet to escape from negative emotions in reality (Kumar and Kumar, 2024). However, overuse of the Internet can isolate individuals from their social relationships, leading to reduced social support and negative emotions (Laconi et al., 2016; Amamou et al., 2017; Dreier et al., 2017). As a result, depression and Internet addiction disorder (IAD) frequently occur simultaneously (Ostinelli et al., 2021). Meanwhile, several studies have reported significant correlations between Internet use and anxiety or stress. A previous study analyzed the relationship between anxiety and Internet addiction among Canadian young people aged 16–19 years. It was reported that 31% of young people were addicted to the Internet, showing a notable positive association between anxiety and Internet addiction (Lavoie et al., 2023). The higher the level of Internet addiction, the higher the degree of anxiety among adolescents (Lavoie et al., 2023; Gundogdu and Eroglu, 2022; Alimoradi et al., 2019), early adult students (Geng et al., 2021; Javaeed et al., 2019; Kayis, 2022) and mature adults (Deb and Roy, 2022; Fekih-Romdhane et al., 2023). Likewise, stress is also closely related to Internet addiction, and individuals with Internet addiction report higher levels of stress than those without Internet addiction (Lei et al., 2020).

In addition to the aforementioned adverse effects of Internet addiction on psychiatric symptoms, studies have also reported a link between Internet addiction and unsatisfactory quality of sleep. Sleep problems can adversely affect well-being and even lead to dysfunction (Awasthi et al., 2020), while impaired sleep quality is connected to a range of adverse outcomes, including impulsivity, depression, anxiety, and even suicidal behavior (Jiang et al., 2022). Internet addiction has an adverse impact on individuals’ sleep quality (Masaeli and Farhadi, 2021; Stankovic et al., 2021; Wang et al., 2021). For example, Gundogdu and Eroglu (2022) found that adolescents with higher Internet (Gundogdu and Eroglu, 2022) addiction scores, as measured by the Pittsburgh Sleep Quality Index (PSQI), were more likely to develop sleep disorders. A meta-analysis in 2019 found a negative correlation between internet addiction and sleep quality in all age groups (Alimoradi et al., 2019), but only sleep duration for sleep disorders has been estimated. The PSQI is a questionnaire used to diagnose sleep disorders, sleep insomnia, and other problems related to sleep quality. It can comprehensively assess the association between Internet addiction and sleep quality. Therefore, this meta-analysis aimed to explore the association between the scores of PSQI and Internet addiction.

Despite the rapid development of the Internet, there is no systematic review and meta-analysis of the correlation of Internet addiction with psychiatric symptom levels and sleep disorders. Furthermore, due to the COVID-19 outbreak, there has been an increase in psychological problems and more time spent using Internet (Deb and Roy, 2022; Zhao et al., 2023). Although there has been a previous meta-analysis (2009–2018) on the relationship between Internet addiction and sleep disorders (Alimoradi et al., 2019), it is unclear whether this association has changed in recent years. Our study could be helpful for future interventions with Internet addiction, psychiatric symptom levels, and sleep disorders.

## 2 Methods

This study was conducted according to the Preferred Reporting Items for Systematic Reviews and Meta-Analyses (PRISMA). This meta-analysis was registered in the International Prospective Register of Systematic Reviews (PROSPERO, CRD42023408958).

### 2.1 Search strategy

Relevant literature was searched in four databases, Web of Science, PubMed, Embase, and Cochrane Library, from January 2018 to June 2023, respectively. We combined the Medical Subject Headings (MeSH) and their free words as the search strategy. MeSH terms included “Internet addiction,” “Psychological distress,” “depression,” “anxiety” and “sleep disturbances.” The detailed search strategy is illustrated in Supplementary Table S1.

### 2.2 Inclusion and exclusion criteria

The inclusion criteria for this study were as follows: (a) studies assessing the association between Internet addiction and anxiety, depression, stress, or sleep quality; (b) studies reported in English; and (c) research guidelines, and studies published between January 2018 and June 2023.

The following studies were excluded: (a) conference abstracts, reviews/meta-analyses, guidelines, and letters; (b) publications with poor quality; and (c) studies with incomplete or erroneous data that could not be combined.

### 2.3 Study selection and data extraction

Two researchers (Yuanlin Sun and Zhen Wang) independently screened studies and extracted data. The retrieved studies were into EndNote 20 to eradicate duplicate publications. We then excluded studies that did not match the eligibility criteria after checking the titles and abstracts. The entire texts of the remaining studies were examined to identify eligible studies. Data extracted from eligible studies included first author, year of publication, geographic location, study design, sample size, average age and gender ratio of participants, and outcome measures such as instrumental coefficients measuring levels of anxiety, depression, impulsivity, and sleep quality. Disagreements were settled by discussion with a third researcher (Tianzhi Liu).

### 2.4 Quality assessment

Two researchers (Yuanlin Sun and Zhen Wang) independently evaluated the methodological quality of the included studies Using the Newcastle-Ottawa Scale (NOS) (Stang, 2010). The quality assessment involves the following domains: randomized sequence generation, allocation concealment, blinding of participants and researchers during the intervention, as well as the integrity of outcome data. The total NOS score ranges from 0 to 10. Typically, a score of greater than 5 indicates a high-quality study. The results were cross-checked, and any discrepancies were resolved through discussion with a third researcher (Tianzhi Liu).

### 2.5 Statistical analysis

The present study employed the inverse variance method to calculate the pooled Pearson’s correlation coefficients, odds ratios (ORs), and mean difference for the association of Internet addiction with anxiety, depression, stress, and sleep quality. According to the study by Zhang et al. (2021), variance-stabilized correlation coefficients were obtained by converting the Pearson correlation coefficients to Fisher Z-scores before pooling the estimates. Heterogeneity across the studies was assessed using the I^2^ statistics, where an *I*^2^ > 50% indicated substantial heterogeneity. If *I*^2^ < 50%, the pooled Pearson’s correlation coefficient was computed using the random-effects model. Otherwise, a fixed-effects model was employed. To identify the source of heterogeneity, sensitivity analysis and subgroup analyses were conducted. Sensitivity analysis was used to examine the reliability of the results, in which we assessed the effect of individual studies on the pooled correlation coefficients by excluding one study at a time. Subgroup analyses were performed by the geographic location of the studies, the mean age of participants, and the IAD measurement tool.

## 3 Results

### 3.1 Search results

**Figure 1:**
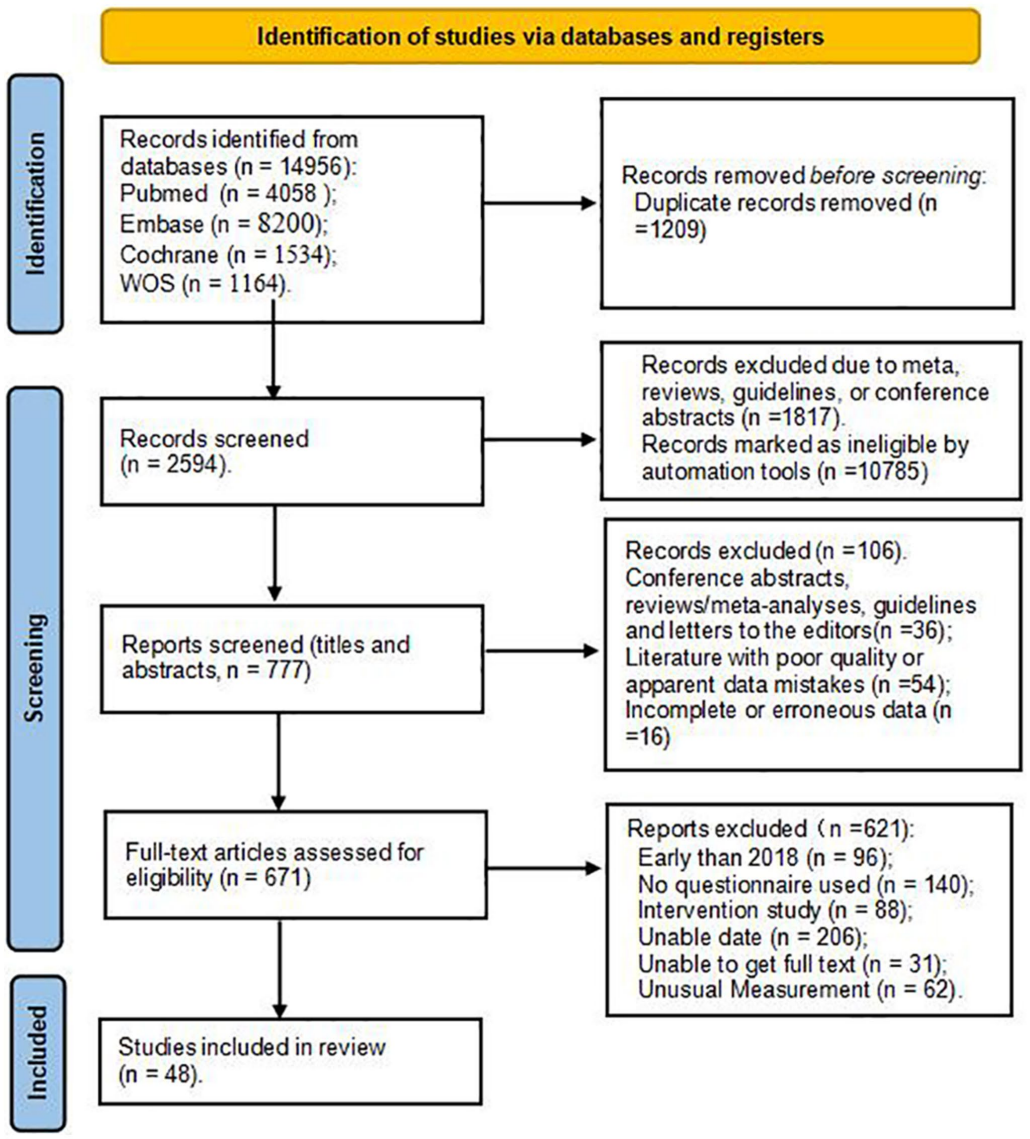
The search process according to the PRISMA flowchart.

The detailed process of study selection is shown in Figure 1. Initially, 29,912 potentially related papers were retrieved. After removing 1,209 duplicate articles, we screened the titles and abstracts and excluded 12,602 studies. Then, the full texts of the remaining 671 articles were examined, and 621 articles were removed because the data was unavailable, unusual measurement tools were used, or they were published before 2018. Finally, 48 studies were included.

### 3.2 Characteristics of the included studies

It is noteworthy that during the data extraction process, baseline data from longitudinal studies were extracted to ensure the comparability, and therefore this study was considered as a cross-sectional study. The 48 eligible studies were published from 2018 to 2023. Among them, 20 studies were conducted in Asia, 10 in Europe, and 4 in South America. There were a total of 35,684 participants. The sample size ranged from 11 to 362, and the average age ranged from 50 to 72 years old. Almost all of the included studies had adequate sample sizes. One study in Singapore had the smallest sample size of 41 participants, while one study in China had the largest sample size of 7,985 participants. In addition, these studies were conducted in 19 countries, and most of them were conducted in China (*n* = 19), followed by Turkey (*n* = 6) and Iran or India (*n* = 3). Young’s Internet Addiction Test (IAT) was the most commonly used tool (*n* = 17) to assess IA status, while the Pittsburg Sleep Quality Index (PSQI) was the most commonly used tool to evaluate sleep problems (Table 1).

**Table 1:** The characteristics of studies included in this meta-analysis.

Author	Year	Nation	*N*	Age	Tools for measuring psychiatric symptom levels and sleep	Tools for measuring Internet addiction
Awasthi et al.	2020	Uttarakhand	221	≥ 18	PSQI	IAT
Azhari et al.	2022	Singapore	41	≤ 18	DASS-21	SMD
Aziz et al.	2018	Klang Valley	199	≤ 18	DASS-21	IAT
Bazrafshan et al.	2019	Iran	119	≥ 18	BDI	IAT
Bisen et al.	2020	India	1,600	≥ 18	BDI	IAT
Chi et al.	2019	China	522	≤ 18	CESD	IAT
Deb et al.	2022	India	258	≥ 18	DASS-21	IAT
Dib et al.	2021	Lebanon	1810	≤ 18	ADRS	IAT
Fekih-Romdhane et al.	2022	Tunis	114	≥ 18	DASS-21	IAT
Ge et al.	2023	Arab Emirates	421	≤ 18	CESD	SAS
Geng et al.	2021	China	355	≥ 18	DASS-21	SAS
Gundogdu et al.	2022	Turkey	244	≤ 18	SCARED/PSQI	PIUS
Gupta et al.	2021	India	292	≥ 18	PHQ-9/PSQI	IAT
Haand et al.	2020	Afghanistan	329	≥ 18	CESD	IAT
Haddad et al.	2021	Lebanon	1,103	≤ 18	ADRS	IAT
Hsieh et al.	2018	Taiwan	500	≥ 18	BDI	IAT
Javaeed et al.	2019	Kashmir	210	≥ 18	DASS-21	IAT
Jiang et al.	2022	China	2,688	≥ 18	SDS/PSQI	IAT
Karakose et al.	2022	Turkey	332	≥ 18	DASS-21	IAT
Kayis et al.	2022	Turkey	110	≥ 18	DASS-21	SAS
Kim et al.	2019	South Korea	4,521	≤ 18	BDI	IAT
Kożybska et al.	2022	Poland	538	≥ 18	BDI	IAT
Kumar et al.	2018	Indian	384	≥ 18	BDI	IAT
Lavoie et al.	2023	Canada	2,883	≤ 18	SCARED	IAT
Lebni et al.	2020	Iran	166	≥ 18	GHQ	IAT
Lee et al.	2022	South Korea	1,155	≥ 18	PHQ-9	IAT
Lei et al.	2020	Malaysia	574	≥ 18	DASS-21	SAS
Mamun et al.	2019	Bangladesh	405	≥ 18	DASS-21	IAT
Mamun et al.	2020	Bangladesh	605	≥ 18	GHQ	IAT
Masaeli et al.	2021	Iran	298	≥ 18	PSQI	IAT
Paudel et al.	2021	Nepal	494	≥ 18	BDI	IAT
Peterka-Bonetta et al.	2019	China	133	≥ 18	BDI	IAT
Przepiorka et al.	2019	Poland	718	≤ 18	CESD	IAT
Saikia et al.	2019	Kamrup	416	≤ 18	DASS-21	IAT
Sami et al.	2018	Israel	631	≤ 18	PHQ	IAT
Sayed et al.	2022	Egypt	808	≥ 18	DASS-21	IAT
Stankovic et al.	2021	Germen	92	≥ 18	PSQI	IAT
Tian et al.	2021	China	1,200	≤ 18	CESD	IAT
Vally et al.	2020	Arab Emirates	697	≥ 18	CESD	PIUQ
Vally et al.	2019	China	706	≥ 18	CESD	PIUQ
Wang et al.	2020	China	1,087	≤ 18	CESD	GPIUS
Wang et al.	2021	China	1,040	≥ 18	PSQI	IAT
Yi et al.	2021	China	1,545	≤ 18	SDS	IAT
Yuan et al.	2021	China	1809	≥ 18	DASS-21	IAT
Yücens et al.	2018	Turkish	392	≥ 18	BDI	IAT
Zhang et al.	2021	China	734	≤ 18	CESD	IAT
Zhao et al.	2023	China	7,958	≤ 18	SCARED/CESD	IAT
Zhao et al.	2022	China	904	≤ 18	CESD	YDQ

PSQI, Pittsburg Sleep Quality Index; DASS, The Depression Anxiety Stress Scale; BDI, Beck Depression Inventory; CESD, Center for Epidemiologic Studies Depression Scale; ADRS, Aphasic Depression Rating Scale; SCARED, The Screen for Child Anxiety Related Emotional Disorders; PHQ-9, Patient Health Questionnaire-9; SDS, Self-Rating Depression Scale; GHQ, General Health Questionnaire; IAT, Internet Addiction Test; SMD, Social Media Dependency Questionnaire; SAS, Smartphone Addiction Scale; GPIUS, Generalized Pathological Internet Use Scale; YDQ, Young’s Diagnostic Questionnaire.

### 3.3 Quality assessment

All 48 studies were of high quality (NOS scores greater than 5) according to the NOS scores. The objectives, variables and measurements were clearly defined, and well-established criteria were used in all studies.

### 3.4 Meta-analysis of the relationship between internet addiction and depression

**Figure 2:**
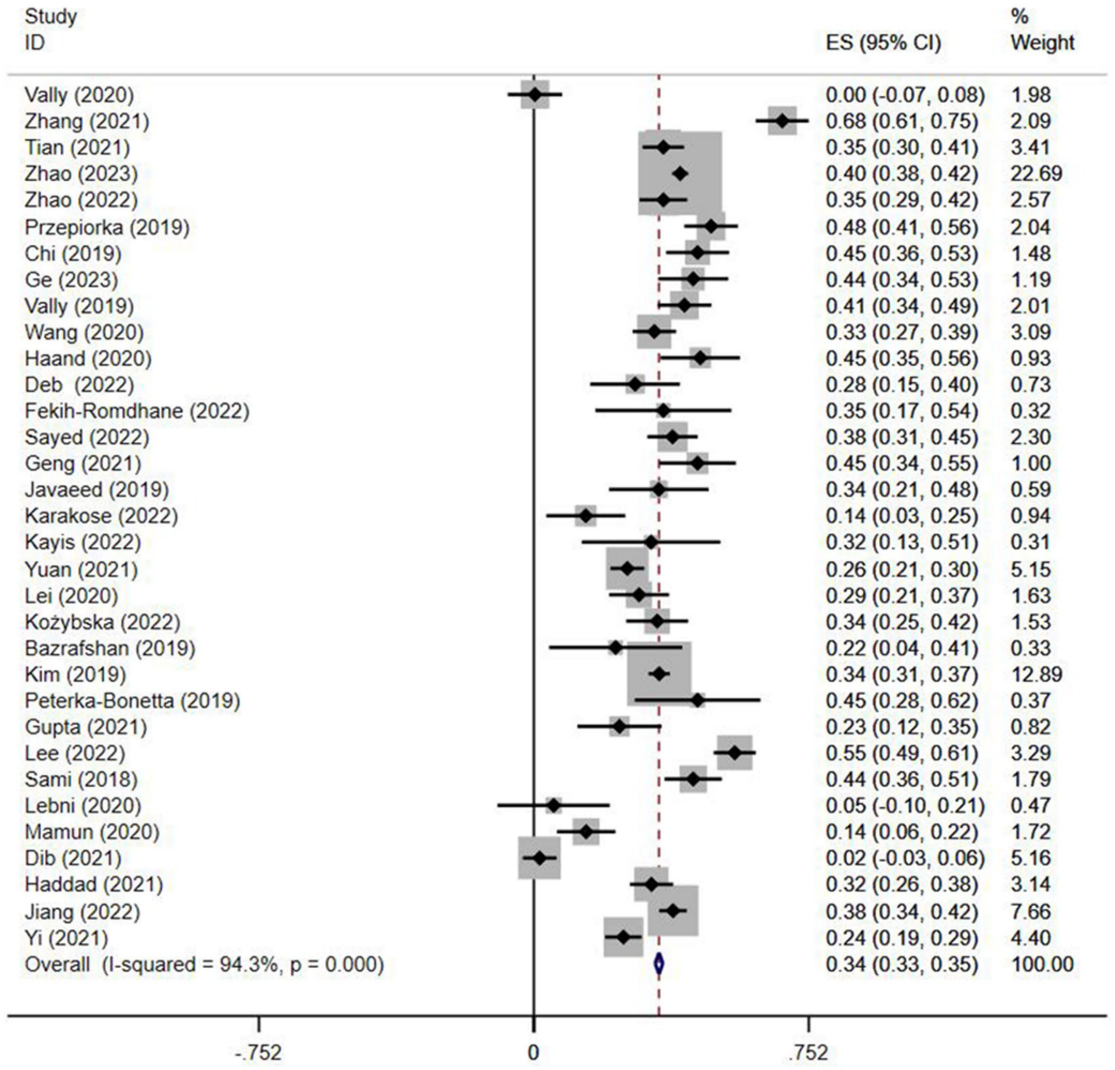
Forest plot of the relationship between depression and Internet addiction.

The relationship between Internet addiction and depression was reported in 33 included studies (Geng et al., 2021; Sayed et al., 2022; Chi et al., 2019; Ge et al., 2023; Yuan et al., 2021; Javaeed et al., 2019; Kayis, 2022; Alimoradi et al., 2019; Deb and Roy, 2022; Fekih-Romdhane et al., 2023; Jiang et al., 2022; Bazrafshan et al., 2019; Gupta et al., 2021; Haddad et al., 2021; Karakose et al., 2022; Kim et al., 2019; Kożybska et al., 2022; Lebni et al., 2020; Lee and Shin, 2022; Peterka-Bonetta et al., 2019; Przepiorka et al., 2019; Sami et al., 2018; Tian et al., 2021; Vally, 2019; Vally et al., 2020; Wang et al., 2020; Yi and Li, 2021; Zhang et al., 2021; Zhao et al., 2022). Because of the substantial heterogeneity between studies (*I*^2^ = 94.3%), the data were pooled using a random-effects model. The results unraveled a significant association between Internet addiction and depression (*r*: 0.39, 95%CI: 0.34–0.45, *p* < 0.001, Figure 2). Three studies (Bisen and Deshpande, 2020; Paudel et al., 2021; Yücens and Üzer, 2018) reported the mean difference in depression scores between Internet addiction and non-Internet addiction groups, and significant differences were found (SMD = 1.34, 95%CI = 0.81–1.86, *p* < 0.001, Supplementary Figure S1). Five studies (Kumar et al., 2018; Aziz et al., 2018; Hsieh et al., 2018; Mamun et al., 2019; Saikia et al., 2019) estimated OR values, and the overall pooled OR was 0.86 (95%CI: 0.46–1.26, *p* < 0.001, Supplementary Figure S2). No publication bias was observed in the funnel plot (Supplementary Figure S3) and Egger’s test (*p* = 0.595).

Subgroup analyses revealed that geographic location and the measurement tool had a significant effect on the pooled correlation coefficients for the association between IAD and depression (Table 2). The association between IAD and depression was not significant in the Turkish (*p* = 0.109) and Irish (*p* = 0.163) populations as well as in studies using BDI (*p* = 0.378) and the GHQ (*p* = 0.314). However, age had no markedly impact on the association between IAD and depression.

**Table 2:** The subgroup analyses revealed the summary correlation coefficient between IAD and depression.

Subgroup	Numbers of studies	*r*	95%CI	*p*	*I* ^2^
Age					
< 18	13	0.35	0.34–0.36	0.000	96.5%
≥ 18	20	0.32	0.31–0.34	0.000	91.2%
Geographic location					
Arab Emirates	2	0.17	0.11–0.22	0.000	98.0%
China	13	0.38	0.36–0.39	0.000	91.8%
Poland	2	0.42	0.37–0.48	0.010	85.0%
Turkey	3	0.19	0.09–0.28	0.109	61.2%
Iran	3	0.12	0.01–0.24	0.163	48.7%
South Korea	2	0.44	0.24–0.65	0.000	97.4%
Lebanon	2	0.13	0.10–0.17	0.000	98.4%
Questionnaires					
CESD	10	0.39	0.38–0.41	0.000	94.5%
DASS-21	15	0.30	0.27–0.33	0.001	68.6%
BDI	9	0.34	0.32–0.37	0.378	3.0%
PHQ	3	0.47	0.43–0.51	0.000	91.7%
GHQ	2	0.12	0.05–0.19	0.314	1.4%
ADRS	2	0.13	0.10–0.17	0.000	98.4%
SDS	2	0.33	0.30–0.36	0.000	94.4%

PSQI, Pittsburg Sleep Quality Index; DASS, The Depression Anxiety Stress Scale; BDI, Beck Depression Inventory; CESD, Center for Epidemiologic Studies Depression Scale; ADRS, Aphasic Depression Rating Scale; SCARED, The Screen for Child Anxiety Related Emotional Disorders; PHQ-9, Patient Health Questionnaire-9; SDS, Self-Rating Depression Scale; GHQ, General Health Questionnaire.

### 3.5 Meta-analysis of the correlation between internet addiction and anxiety

**Figure 3:**
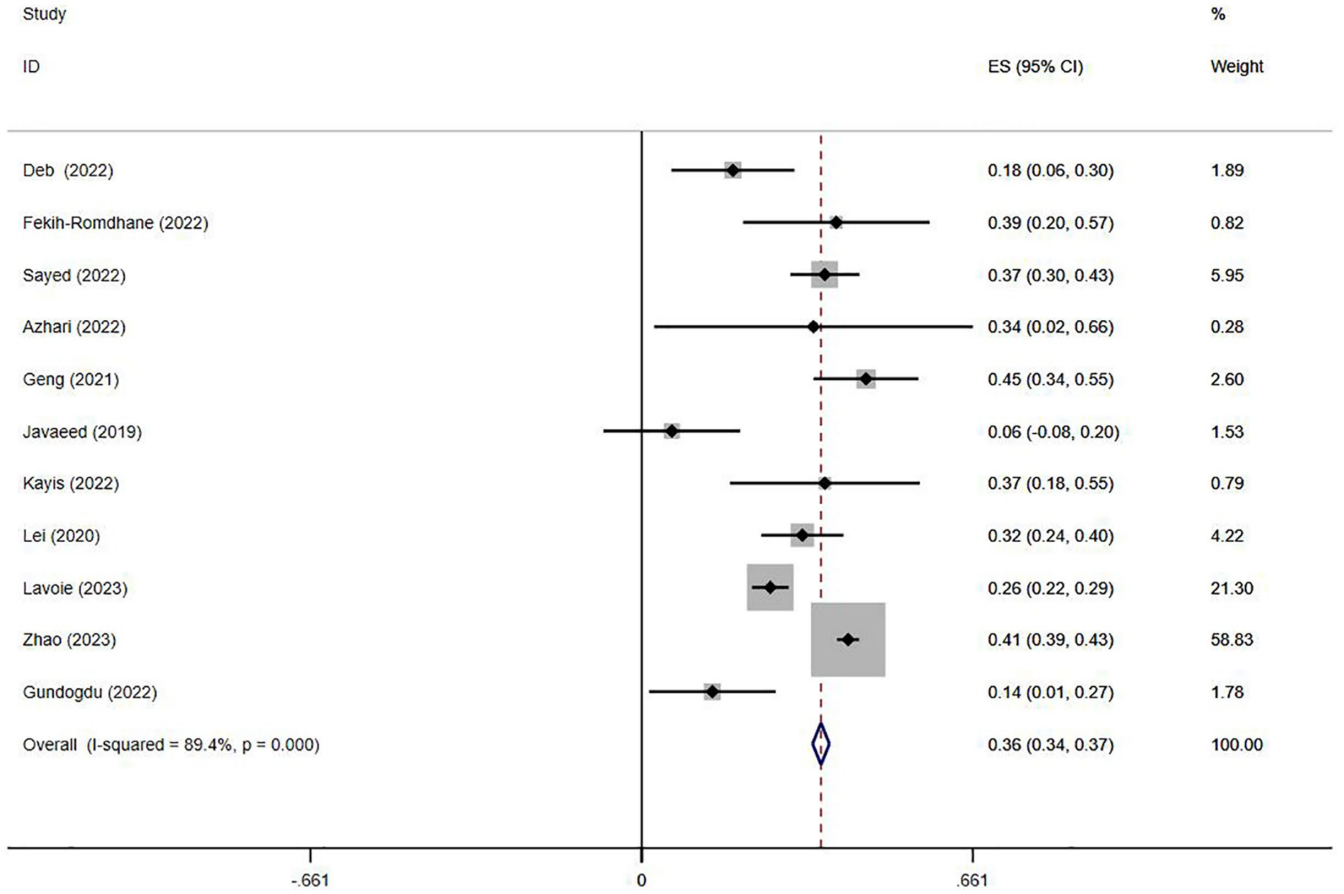
Forest plot of the relationship between anxiety and Internet addiction.

Eleven studies (Geng et al., 2021; Sayed et al., 2022; Javaeed et al., 2019; Kayis, 2022; Lavoie et al., 2023; Gundogdu and Eroglu, 2022; Alimoradi et al., 2019; Deb and Roy, 2022; Fekih-Romdhane et al., 2023; Lei et al., 2020; Aziz et al., 2018) reported the association between Internet addiction and anxiety. The heterogeneity for anxiety was high (*I*^2^ = 89.4%, *p* < 0.001). The pooled r value was 0.30 (95%CI: 0.23–0.37, *p* < 0.001, Figure 3). Three studies reported OR values (Aziz et al., 2018; Mamun et al., 2019; Saikia et al., 2019), resulting in a pooled OR of 0.90 (95%CI = 0.29–1.52, *p* = 0.133, Supplementary Figure S4). The funnel plot (Supplementary Figure S5) and the Egger’s Test (*p* = 0.162) indicated no publication bias.

Subgroup analyses were conducted only based on participants’ age and measurement tools due to insufficient information. The findings of the subgroup analyses suggested that age and measurement tools did not obviously affect the association between Internet addiction and anxiety (*I*^2^ > 50%, Table 3).

**Table 3:** The subgroup analyses revealed the summary correlation coefficient between IAD and anxiety.

Subgroup	Numbers of studies	*r*	95%CI	*p*	*I* ^2^
Age					
< 18	5	0.37	0.35–0.38	0.000	93.7%
≥ 18	6	0.30	0.25–0.35	0.000	79.8%
Questionnaires					
DASS-21	8	0.32	0.28–0.36	0.000	74.1%
SCARED	3	0.37	0.35–0.38	0.000	96.8%

### 3.6 Meta-analysis of the correlation between internet addiction and stress

**Figure 4:**
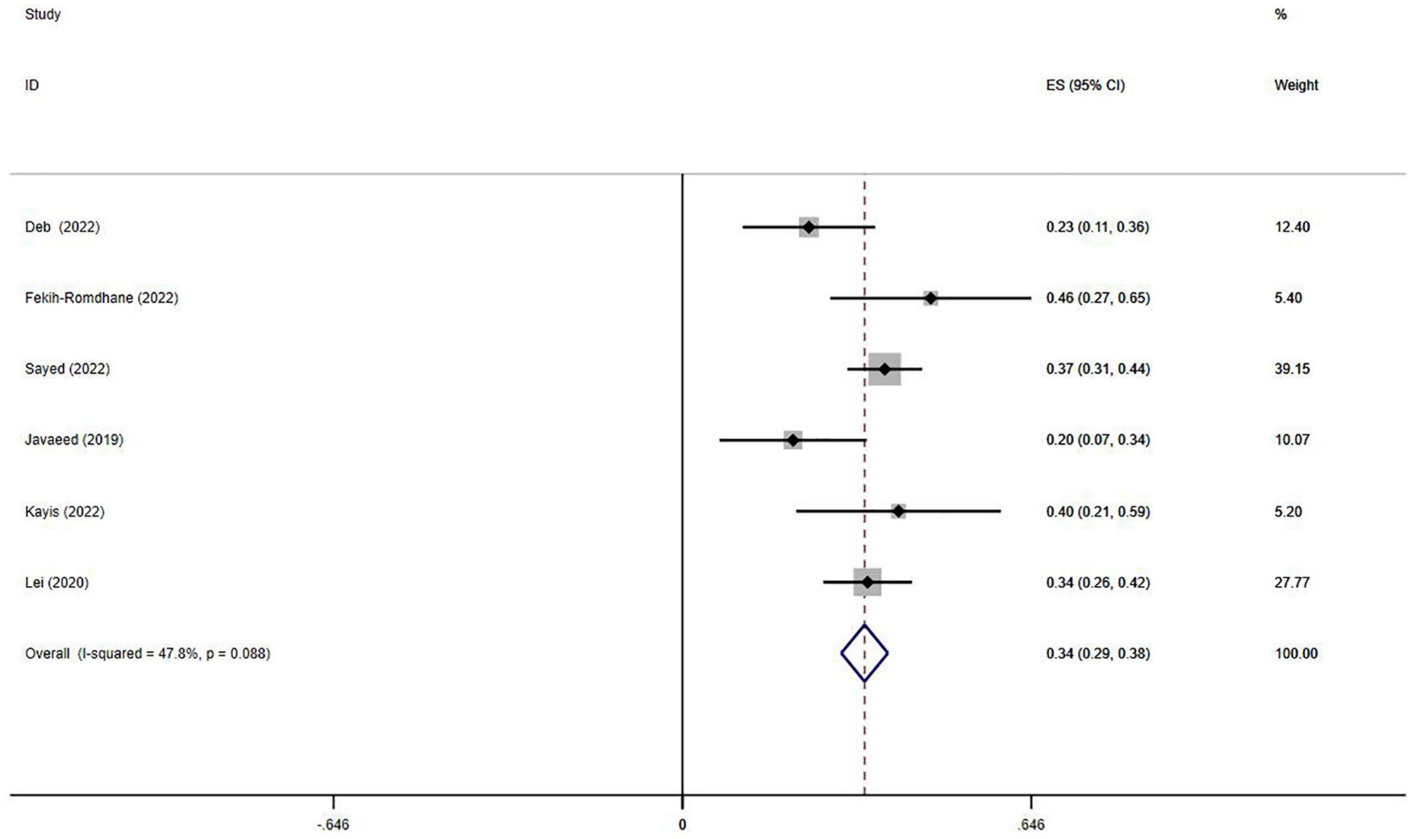
Forest plot of the relationship between stress and Internet addiction.

**Figure 5:**
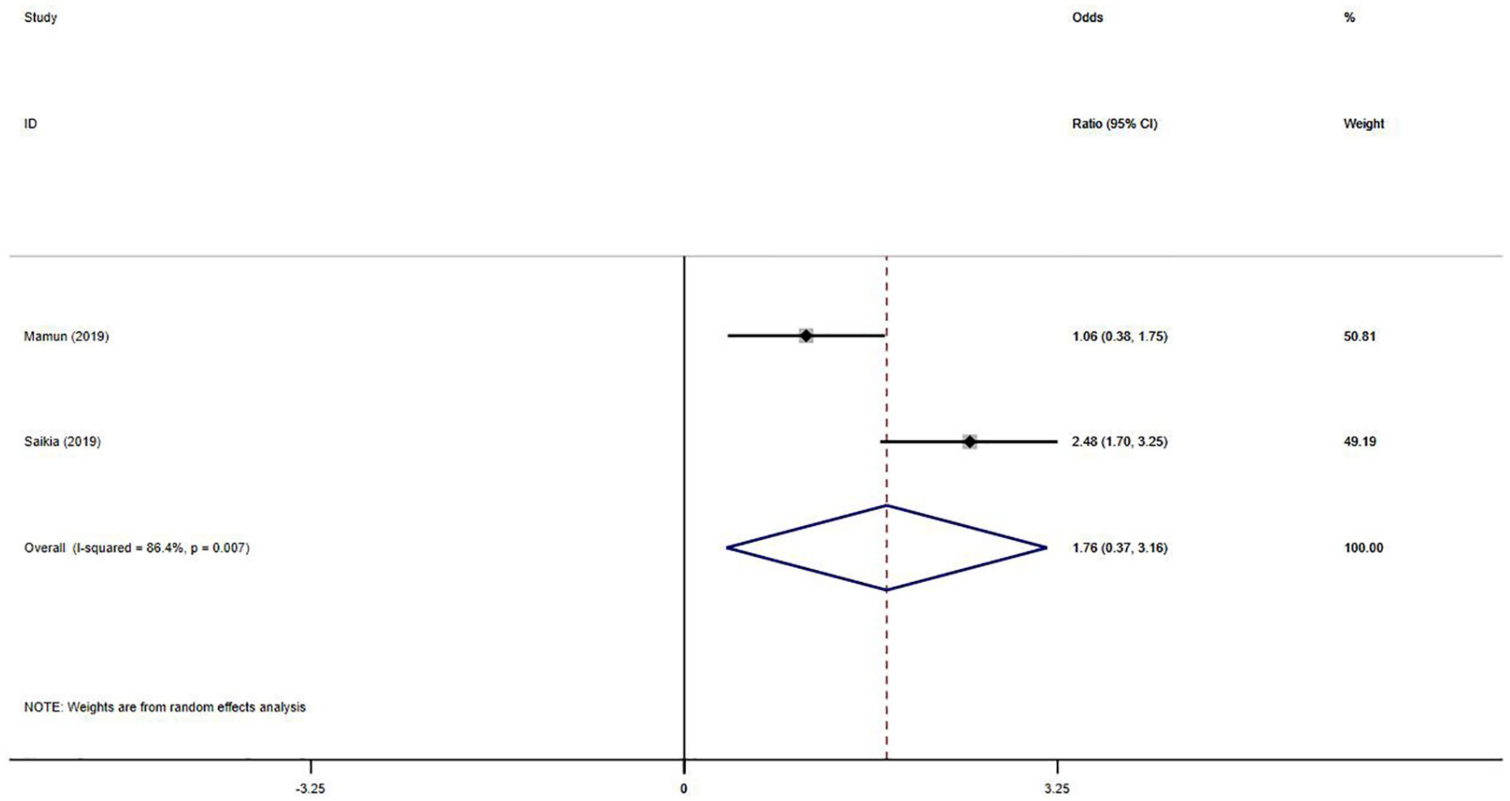
Forest plot of the pooled OR for the association between stress and Internet addiction.

Six studies (Sayed et al., 2022; Javaeed et al., 2019; Kayis, 2022; Deb and Roy, 2022; Fekih-Romdhane et al., 2023; Lei et al., 2020) explored the correlation between Internet addiction and stress. The pooled r value was 0.34 (95%CI: 0.29–0.38, *p* = 0.088, *I*^2^ = 47.8%, Figure 4). Two studies reported OR values (Mamun et al., 2019; Saikia et al., 2019), and the pooled OR was 1.76 (95%CI: 0.37–3.16, *p* = 0.007, Figure 5). No publication bias was observed (Egger’s *p* = 0.778).

### 3.7 Meta-analysis of the relation between internet addiction and sleep quality

**Figure 6:**
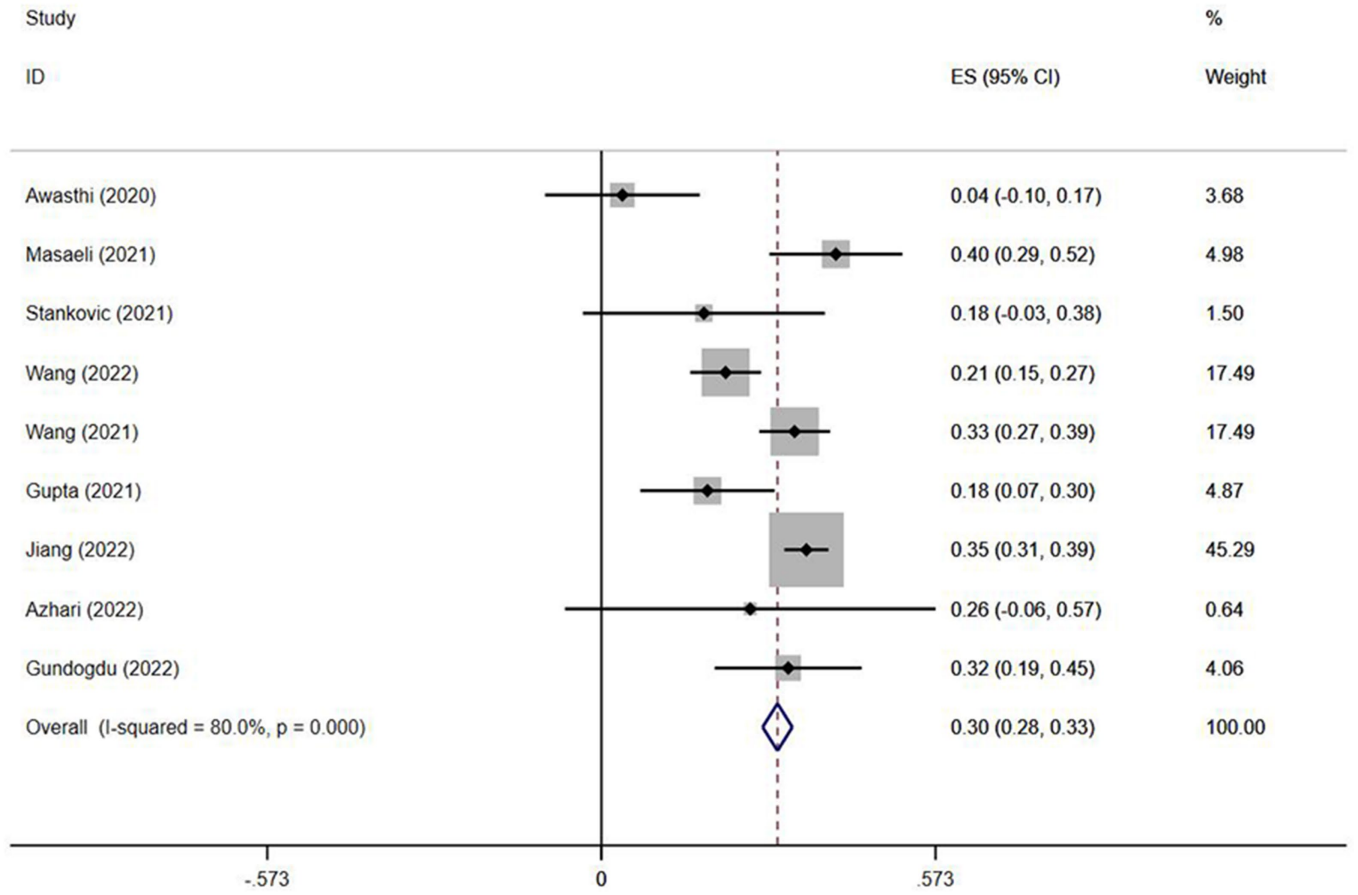
Forest plot of the relationship between sleep quality and Internet addiction.

Nine studies (Gundogdu and Eroglu, 2022; Awasthi et al., 2020; Jiang et al., 2022; Masaeli and Farhadi, 2021; Stankovic et al., 2021; Wang et al., 2021; Gupta et al., 2021; Wang et al., 2020; Azhari et al., 2022) investigated the association between Internet addiction and sleep quality. The pooled r-value was 0.26 (95%CI: 0.19–0.33, *p* < 0.001, *I*^2^ = 80.0%). Moreover, the results of the subgroup analysis showed that age did not affect the correlation between IAD and sleep quality (Figure 6). The visual funnel plots (Supplementary Figure S6) and the Egger’s Test (*p* = 0.192) showed no publication bias.

### 3.8 Sensitivity analyses

Sensitivity analyses were conducted to assess the reliability of the study results by systematically removing one individual study at a time and recalculating the pooled correlation coefficients. The sensitivity analyses for depression, anxiety, stress, and sleep quality showed minimal changes in the meta-analysis results, suggesting that our results were stable.

## 4 Discussion

This study investigated the association of IAD with depression, anxiety, sleep quality, and stress. The results of the meta-analyses showed that IAD was weakly to moderately positively correlated with depression, anxiety, sleep quality, and stress (*r*: 0.26–0.39, pooled OR: 0.86, 0.9, 1.76, respectively), and the mean difference in depression scores between Internet addiction and non-internet addiction groups was 1.34.

The results of the meta-analysis indicated a significant relationship between IAD and depression, which is consistent with previous findings (Bazrafshan et al., 2019; Lee and Shin, 2022). According to subgroup analyses, the relationship between Internet addiction and depression remained significant in the Arab Emirates, China, Poland, South Korea, and Lebanon, but not in Türkiye and Ireland. This difference may be caused by differences in culture and social rules among different countries. In countries with collectivist cultures such as the United Arab Emirates and China, young people reconstruct their identities through virtual spaces, turning the Internet into a “haven” from the pressure of reality. In countries undergoing social transition, such as Poland and South Korea, economic pressures and time poverty have led to “compensatory” dependence, so that virtual achievements become substitutes for real-world deficiencies. On the other hand, Catholic culture in Ireland strengthens behavioral self-discipline through rituals such as confession, and community activities provide offline meaning networks. Islamic culture in Türkiye turns technological tools into means of production, and traditional coffee-house culture maintains embodied social interactions, forming a natural immunity to Internet addiction. At the same time, it may also be due to diversified measurement instruments. No significant differences are observed between BDI and GHQ, which are mainly used in Irish and Turkish studies.

Similarly, there was a significant correlation between IAD and anxiety, consistent with a previous study (Sayed et al., 2022; Wong et al., 2020; Fekih-Romdhane et al., 2023; Lebni et al., 2020). Because of insufficient data, subgroup analyses were only performed according to age and measurement instruments. It was shown that age and measurement instruments did not affect the correlation between IAD and anxiety. However, there was no significant association between IAD and stress. This may be because, unlike anxiety and depression, individuals experiencing stress may adopt alternative stress relief strategies, which potentially reduce their susceptibility to developing Internet addiction.

The current study offered evidence that psychiatric symptom levels and Internet addiction were significantly interrelated before and during the outbreak of the COVID-19 pandemic. People who constantly use the Internet and become overly addicted to the virtual world are more likely to suffer from depression. After Internet addiction, they tend to become more isolated from reality and social relationships, leading to a vicious cycle. Therefore, if individuals can reduce their Internet addiction and increase real-world activities, their emotions may be more positive. The reverse is also true, if an individual’s psychological symptoms are reduced, their Internet addiction behavior may also be reduced. Therefore, in practical applications, a multi-dimensional approach can be adopted for the comorbidity of IAD and psychological problems.

Additionally, we explored the relationship between Internet addiction and sleep quality. The results indicate that there is indeed a correlation between Internet addiction and sleep quality. Subgroup analysis found that Internet addiction was significantly associated with sleep quality in both adults (age ≥ 18 years) and children (age ≤ 18 years). To the best of our knowledge, although an increasing number of studies investigated the relationship between Internet addiction and sleep (including duration and quality of sleep), the latest meta-analysis on IAD and sleep was published in 2019. Moreover, the epidemic of COVID-19 in recent years has transformed our lifestyles. The frequency of using the Internet has accordingly increased significantly; hence, the evidence in recent years needs to be summarized. Moreover, in 2019, a study on the relationship between sleep duration and Internet addiction found that factors such as sleep duration, dreaminess during sleep, and daytime mental state were also crucial in evaluating sleep quality. Nevertheless, no systematic review and meta-analysis has been conducted to summarize these findings. There are several explanations for the positive correlation between Internet addiction and sleep quality (Li et al., 2020). Psychologically, excessive use of the Internet before bedtime will lead to higher psychological arousal levels, which may compromise sleep quality. Physiologically, electronic screens emit electromagnetic fields that can impact the secretion of melatonin in the body, which in turn affects the quality of sleep. Subgroup analysis by age revealed significant differences in the association between IAD and sleep quality. In individuals above 18 years old, Internet addiction and sleep quality are significantly correlated, whereas in those below 18 years old, Internet addiction and sleep quality were not significantly correlated. These results indicate that age is the primary factor affecting the relationship between IAD and sleep quality. This may be because adolescents are often supervised by parents or schools, and the mandatory schedule may offset the direct effects of Internet addiction on sleep; adults are more likely to form bad habits such as “staying up late” by scheduling their time.

This study provides valuable evidence about the relationship between Internet addiction, psychiatric symptom levels, and sleep disorders. Nevertheless, there are several limitations. Firstly, most of the results are self-reported by the participants through questionnaires, and as such, may be affected by subjective factors. Secondly, there was a great degree of heterogeneity among the studies. However, due to the limited number of the included studies, the source of heterogeneity may not be fully explored. Thirdly, it is imperative to study the longitudinal association of Internet addiction with psychiatric symptom levels and sleep disorders. Inadequate literature currently restrains us from doing the analysis. Therefore, more high-quality studies are warranted to confirm our findings.

## 5 Conclusion

Our results reveal that Internet addiction is associated with depression, anxiety, and stress, and compromises the sleep quality of both adults and adolescents. However, further research is required to investigate the potential negative effects of internet addiction. Encouraging people to use the Internet in a controlled manner (for example, not to use the Internet so excessively to lose social interaction, or to reduce Internet use before bedtime) can help to alleviate negative consequences. Future research should investigate the direction of this association and determine whether Internet addiction has a greater impact on sleep in specific populations.

## Data Availability

The original contributions presented in the study are included in the article/Supplementary material, further inquiries can be directed to the corresponding author.
