# Supplementary material for: Association of Internet addiction with psychiatric symptom levels and sleep disorders: a systematic review and meta-analysis
Source: Front Psychol. 2025 Apr 17;16:1573058. doi: 10.3389/fpsyg.2025.1573058 (PMC12043566; doi:10.3389/fpsyg.2025.1573058)

**Table S1 Literature search strategy**

**1.Pubmed**

| Search number | Query | Results |
| --- | --- | --- |
| #1 | "Internet Addiction Disorder"[Mesh] | 31 |
| #2 | (((((((((((((((((((((((((((((((((Internet addiction disorders[Title/Abstract]) OR (Internet gaming disorder[Title/Abstract])) OR (Internet use disorder[Title/Abstract])) OR (excessive internet use[Title/Abstract])) OR (problematic internet use[Title/Abstract])) OR (sexual preoccupations[Title/Abstract])) OR (Social excessive email[Title/Abstract])) OR (Social excessive text[Title/Abstract])) OR (Internet dependency[Title/Abstract])) OR (computer addiction[Title/Abstract])) OR (Addiction Disorder, Internet[Title/Abstract])) OR (Social Media Addiction[Title/Abstract])) OR (Addiction, Social Media[Title/Abstract])) OR (Addictions, Social Media[Title/Abstract])) OR (Addiction, Smartphone[Title/Abstract])) OR (Addictions, Smartphone[Title/Abstract])) OR (Disorders, Internet Gaming[Title/Abstract])) OR (compulsive internet usage[Title/Abstract])) OR (compulsive internet use[Title/Abstract])) OR (excessive internet usage[Title/Abstract])) OR (excessive internet use[Title/Abstract])) OR (internet addiction disorder[Title/Abstract])) OR (internet dependence[Title/Abstract])) OR (internet dependency[Title/Abstract])) OR (internet over-use[Title/Abstract])) OR (internet overusage[Title/Abstract])) OR (internet overuse[Title/Abstract])) OR (online addiction[Title/Abstract])) OR (online media addiction[Title/Abstract])) OR (pathologic internet use[Title/Abstract])) OR (pathological internet usage[Title/Abstract])) OR (pathological internet use[Title/Abstract])) OR (problematic internet usage[Title/Abstract])) OR (problematic internet use[Title/Abstract]) | 64 |
| #3 | ("Internet Addiction Disorder"[Mesh]) OR ((((((((((((((((((((((((((((((((((Internet addiction disorders[Title/Abstract]) OR (Internet gaming disorder[Title/Abstract])) OR (Internet use disorder[Title/Abstract])) OR (excessive internet use[Title/Abstract])) OR (problematic internet use[Title/Abstract])) OR (sexual preoccupations[Title/Abstract])) OR (Social excessive email[Title/Abstract])) OR (Social excessive text[Title/Abstract])) OR (Internet dependency[Title/Abstract])) OR (computer addiction[Title/Abstract])) OR (Addiction Disorder, Internet[Title/Abstract])) OR (Social Media Addiction[Title/Abstract])) OR (Addiction, Social Media[Title/Abstract])) OR (Addictions, Social Media[Title/Abstract])) OR (Addiction, Smartphone[Title/Abstract])) OR (Addictions, Smartphone[Title/Abstract])) OR (Disorders, Internet Gaming[Title/Abstract])) OR (compulsive internet usage[Title/Abstract])) OR (compulsive internet use[Title/Abstract])) OR (excessive internet usage[Title/Abstract])) OR (excessive internet use[Title/Abstract])) OR (internet addiction disorder[Title/Abstract])) OR (internet dependence[Title/Abstract])) OR (internet dependency[Title/Abstract])) OR (internet over-use[Title/Abstract])) OR (internet overusage[Title/Abstract])) OR (internet overuse[Title/Abstract])) OR (online addiction[Title/Abstract])) OR (online media addiction[Title/Abstract])) OR (pathologic internet use[Title/Abstract])) OR (pathological internet usage[Title/Abstract])) OR (pathological internet use[Title/Abstract])) OR (problematic internet usage[Title/Abstract])) OR (problematic internet use[Title/Abstract])) | 76 |
| #4 | "Psychological Distress"[Mesh] | 76 |
| #5 | ((((((Psychological Distress[Title/Abstract]) OR (Distress, Psychological[Title/Abstract])) OR (Emotional Distress[Title/Abstract])) OR (Distress, Emotional[Title/Abstract])) OR (Emotional Stress[Title/Abstract])) OR (Stress, Emotional[Title/Abstract])) OR (dystress syndrome[Title/Abstract]) | 460 |
| #6 | ("Psychological Distress"[Mesh]) OR (((((((Psychological Distress[Title/Abstract]) OR (Distress, Psychological[Title/Abstract])) OR (Emotional Distress[Title/Abstract])) OR (Distress, Emotional[Title/Abstract])) OR (Emotional Stress[Title/Abstract])) OR (Stress, Emotional[Title/Abstract])) OR (dystress syndrome[Title/Abstract])) | 492 |
| #7 | ((((((((((((((((((((((((((((((((((((((((((((((((((((((((((((((((Depressive Symptoms) OR (Depressive Symptom)) OR (Depression, Emotional)) OR (central depression)) OR (clinical depression)) OR (depressive disease)) OR (depressive disorder)) OR (depressive episode)) OR (depressive illness)) OR (depressive personality disorder)) OR (depressive state)) OR (depressive symptom)) OR (depressive syndrome)) OR (depressive syndrome)) OR (Angst)) OR (Social Anxiety)) OR (Anxieties, Social)) OR (Anxiety, Social)) OR (Hypervigilance)) OR (Nervousness)) OR (Anxiousness)) OR (alarm reaction)) OR (biologic stress)) OR (biological stress)) OR (organismal stress)) OR (physiologic stress)) OR (physiological stresses)) OR (stress reaction)) OR (stress resistance)) OR (stress response)) OR (stress situation)) OR (stress tolerance)) OR (stress, physiological)) OR (Substance Related Disorder)) OR (Substance-use)) OR (Substance Related Disorder)) OR (Related Disorders, Substance)) OR (Drug Use Disorders)) OR (Drug Use Disorder)) OR (Organic Mental Disorders, Substance-Induced)) OR (Abuse, Substance)) OR (Substance Abuses)) OR (Substance Dependence)) OR (Substance Addiction)) OR (Chemical Dependence)) OR (Chemical Dependences)) OR (Dependence, Chemical)) OR (Dependences, Chemical)) OR (Dependence, Drug)) OR (Addiction, Drug)) OR (Prescription Drug Abuse)) OR (Substance Use)) OR (Substance Uses)) OR (Drug Abuse)) OR (Habituation, Drug)) OR (Substance Use Disorders)) OR (Disorder, Substance Use)) OR (attention-deficit)) OR (attention deficit)) OR (attention deficit and disruptive behaviour disorders)) OR (attention deficit disorder)) OR (attention deficit disorder)) OR (Depression)) OR (Anxiety)) OR (Stress) | 20819 |
| #8 | ("Psychological Distress"[Mesh]) OR (((((((((((((((((((((((((((((((((((((((((((((((((((((((((((((((((Depressive Symptoms) OR (Depressive Symptom)) OR (Depression, Emotional)) OR (central depression)) OR (clinical depression)) OR (depressive disease)) OR (depressive disorder)) OR (depressive episode)) OR (depressive illness)) OR (depressive personality disorder)) OR (depressive state)) OR (depressive symptom)) OR (depressive syndrome)) OR (depressive syndrome)) OR (Angst)) OR (Social Anxiety)) OR (Anxieties, Social)) OR (Anxiety, Social)) OR (Hypervigilance)) OR (Nervousness)) OR (Anxiousness)) OR (alarm reaction)) OR (biologic stress)) OR (biological stress)) OR (organismal stress)) OR (physiologic stress)) OR (physiological stresses)) OR (stress reaction)) OR (stress resistance)) OR (stress response)) OR (stress situation)) OR (stress tolerance)) OR (stress, physiological)) OR (Substance Related Disorder)) OR (Substance-use)) OR (Substance Related Disorder)) OR (Related Disorders, Substance)) OR (Drug Use Disorders)) OR (Drug Use Disorder)) OR (Organic Mental Disorders, Substance-Induced)) OR (Abuse, Substance)) OR (Substance Abuses)) OR (Substance Dependence)) OR (Substance Addiction)) OR (Chemical Dependence)) OR (Chemical Dependences)) OR (Dependence, Chemical)) OR (Dependences, Chemical)) OR (Dependence, Drug)) OR (Addiction, Drug)) OR (Prescription Drug Abuse)) OR (Substance Use)) OR (Substance Uses)) OR (Drug Abuse)) OR (Habituation, Drug)) OR (Substance Use Disorders)) OR (Disorder, Substance Use)) OR (attention-deficit)) OR (attention deficit)) OR (attention deficit and disruptive behaviour disorders)) OR (attention deficit disorder)) OR (attention deficit disorder)) OR (Depression)) OR (Anxiety)) OR (Stress)) | 20824 |
| #9 | (("Psychological Distress"[Mesh]) OR (((((((Psychological Distress[Title/Abstract]) OR (Distress, Psychological[Title/Abstract])) OR (Emotional Distress[Title/Abstract])) OR (Distress, Emotional[Title/Abstract])) OR (Emotional Stress[Title/Abstract])) OR (Stress, Emotional[Title/Abstract])) OR (dystress syndrome[Title/Abstract]))) OR (((((((((((((((((((((((((((((((((((((((((((((((((((((((((((((((((Depressive Symptoms) OR (Depressive Symptom)) OR (Depression, Emotional)) OR (central depression)) OR (clinical depression)) OR (depressive disease)) OR (depressive disorder)) OR (depressive episode)) OR (depressive illness)) OR (depressive personality disorder)) OR (depressive state)) OR (depressive symptom)) OR (depressive syndrome)) OR (depressive syndrome)) OR (Angst)) OR (Social Anxiety)) OR (Anxieties, Social)) OR (Anxiety, Social)) OR (Hypervigilance)) OR (Nervousness)) OR (Anxiousness)) OR (alarm reaction)) OR (biologic stress)) OR (biological stress)) OR (organismal stress)) OR (physiologic stress)) OR (physiological stresses)) OR (stress reaction)) OR (stress resistance)) OR (stress response)) OR (stress situation)) OR (stress tolerance)) OR (stress, physiological)) OR (Substance Related Disorder)) OR (Substance-use)) OR (Substance Related Disorder)) OR (Related Disorders, Substance)) OR (Drug Use Disorders)) OR (Drug Use Disorder)) OR (Organic Mental Disorders, Substance-Induced)) OR (Abuse, Substance)) OR (Substance Abuses)) OR (Substance Dependence)) OR (Substance Addiction)) OR (Chemical Dependence)) OR (Chemical Dependences)) OR (Dependence, Chemical)) OR (Dependences, Chemical)) OR (Dependence, Drug)) OR (Addiction, Drug)) OR (Prescription Drug Abuse)) OR (Substance Use)) OR (Substance Uses)) OR (Drug Abuse)) OR (Habituation, Drug)) OR (Substance Use Disorders)) OR (Disorder, Substance Use)) OR (attention-deficit)) OR (attention deficit)) OR (attention deficit and disruptive behaviour disorders)) OR (attention deficit disorder)) OR (attention deficit disorder)) OR (Depression)) OR (Anxiety)) OR (Stress)) | 20935 |
| #10 | (("Internet Addiction Disorder"[Mesh]) OR ((((((((((((((((((((((((((((((((((Internet addiction disorders[Title/Abstract]) OR (Internet gaming disorder[Title/Abstract])) OR (Internet use disorder[Title/Abstract])) OR (excessive internet use[Title/Abstract])) OR (problematic internet use[Title/Abstract])) OR (sexual preoccupations[Title/Abstract])) OR (Social excessive email[Title/Abstract])) OR (Social excessive text[Title/Abstract])) OR (Internet dependency[Title/Abstract])) OR (computer addiction[Title/Abstract])) OR (Addiction Disorder, Internet[Title/Abstract])) OR (Social Media Addiction[Title/Abstract])) OR (Addiction, Social Media[Title/Abstract])) OR (Addictions, Social Media[Title/Abstract])) OR (Addiction, Smartphone[Title/Abstract])) OR (Addictions, Smartphone[Title/Abstract])) OR (Disorders, Internet Gaming[Title/Abstract])) OR (compulsive internet usage[Title/Abstract])) OR (compulsive internet use[Title/Abstract])) OR (excessive internet usage[Title/Abstract])) OR (excessive internet use[Title/Abstract])) OR (internet addiction disorder[Title/Abstract])) OR (internet dependence[Title/Abstract])) OR (internet dependency[Title/Abstract])) OR (internet over-use[Title/Abstract])) OR (internet overusage[Title/Abstract])) OR (internet overuse[Title/Abstract])) OR (online addiction[Title/Abstract])) OR (online media addiction[Title/Abstract])) OR (pathologic internet use[Title/Abstract])) OR (pathological internet usage[Title/Abstract])) OR (pathological internet use[Title/Abstract])) OR (problematic internet usage[Title/Abstract])) OR (problematic internet use[Title/Abstract]))) AND ((("Psychological Distress"[Mesh]) OR (((((((Psychological Distress[Title/Abstract]) OR (Distress, Psychological[Title/Abstract])) OR (Emotional Distress[Title/Abstract])) OR (Distress, Emotional[Title/Abstract])) OR (Emotional Stress[Title/Abstract])) OR (Stress, Emotional[Title/Abstract])) OR (dystress syndrome[Title/Abstract]))) OR (((((((((((((((((((((((((((((((((((((((((((((((((((((((((((((((((Depressive Symptoms) OR (Depressive Symptom)) OR (Depression, Emotional)) OR (central depression)) OR (clinical depression)) OR (depressive disease)) OR (depressive disorder)) OR (depressive episode)) OR (depressive illness)) OR (depressive personality disorder)) OR (depressive state)) OR (depressive symptom)) OR (depressive syndrome)) OR (depressive syndrome)) OR (Angst)) OR (Social Anxiety)) OR (Anxieties, Social)) OR (Anxiety, Social)) OR (Hypervigilance)) OR (Nervousness)) OR (Anxiousness)) OR (alarm reaction)) OR (biologic stress)) OR (biological stress)) OR (organismal stress)) OR (physiologic stress)) OR (physiological stresses)) OR (stress reaction)) OR (stress resistance)) OR (stress response)) OR (stress situation)) OR (stress tolerance)) OR (stress, physiological)) OR (Substance Related Disorder)) OR (Substance-use)) OR (Substance Related Disorder)) OR (Related Disorders, Substance)) OR (Drug Use Disorders)) OR (Drug Use Disorder)) OR (Organic Mental Disorders, Substance-Induced)) OR (Abuse, Substance)) OR (Substance Abuses)) OR (Substance Dependence)) OR (Substance Addiction)) OR (Chemical Dependence)) OR (Chemical Dependences)) OR (Dependence, Chemical)) OR (Dependences, Chemical)) OR (Dependence, Drug)) OR (Addiction, Drug)) OR (Prescription Drug Abuse)) OR (Substance Use)) OR (Substance Uses)) OR (Drug Abuse)) OR (Habituation, Drug)) OR (Substance Use Disorders)) OR (Disorder, Substance Use)) OR (attention-deficit)) OR (attention deficit)) OR (attention deficit and disruptive behaviour disorders)) OR (attention deficit disorder)) OR (attention deficit disorder)) OR (Depression)) OR (Anxiety)) OR (Stress))) | 29 |

**2.Cochrane**

| Search number | Query | Results |
| --- | --- | --- |
| #1 | MeSH descriptor: [Internet Addiction Disorder] explode all trees | 30 |
| #2 | (Internet addiction disorders):ti,ab,kw OR (Internet use disorder):ti,ab,kw OR (excessive internet use):ti,ab,kw OR (problematic internet use):ti,ab,kw | 443 |
| #3 | (Internet Addictions):ti,ab,kw AND (Addictions, Smartphone):ti,ab,kw | 4 |
| #4 | #1 or #2 or #3 | 443 |
| #5 | MeSH descriptor: [Psychological Distress] explode all trees | 424 |
| #6 | (Psychological Distress):ti,ab,kw AND (depression):ti,ab,kw AND (anxiety):ti,ab,kw | 2764 |
| #7 | #5 or #6 | 3089 |
| #8 | #4 and #7 | 8 |
| #9 | MeSH descriptor: [Internet Addiction Disorder] explode all trees | 30 |
| #10 | (Internet addiction disorders):ti,ab,kw OR (Internet gaming disorder):ti,ab,kw OR (Internet use disorder):ti,ab,kw OR (excessive internet use):ti,ab,kw OR (problematic internet use):ti,ab,kw | 1294 |
| #11 | (sexual preoccupations):ti,ab,kw OR (Social excessive email):ti,ab,kw OR (Social excessive text):ti,ab,kw OR (Internet dependency):ti,ab,kw OR (computer addiction):ti,ab,kw | 435 |
| #12 | (Addiction Disorder, Internet):ti,ab,kw OR (Addiction, Social Media):ti,ab,kw OR (Addictions, Social Media):ti,ab,kw OR (Addiction, Smartphone):ti,ab,kw OR (Addictions, Smartphone):ti,ab,kw | 389 |
| #13 | (Disorders, Internet Gaming):ti,ab,kw OR (compulsive internet usage):ti,ab,kw OR (compulsive internet use):ti,ab,kw OR (excessive internet usage):ti,ab,kw OR (excessive internet use):ti,ab,kw | 195 |
| #14 | (internet dependence):ti,ab,kw OR (internet over-use):ti,ab,kw OR (internet overusage):ti,ab,kw OR (internet overuse):ti,ab,kw OR (online addiction):ti,ab,kw | 583 |
| #15 | (online media addiction):ti,ab,kw OR (pathologic internet use):ti,ab,kw OR (pathological internet usage):ti,ab,kw OR (pathological internet use):ti,ab,kw OR (problematic internet usage):ti,ab,kw | 122 |
| #16 | #9 or #10 or #11 or #12 or #13 or #14 or #15 | 2234 |
| #17 | (Psychological Distress):ti,ab,kw OR (Emotional Distress):ti,ab,kw OR (Emotional Stress):ti,ab,kw OR (dystress syndrome):ti,ab,kw OR (Depression):ti,ab,kw | 107052 |
| #18 | (Depressive Symptoms):ti,ab,kw OR (Depressive Symptom):ti,ab,kw OR (Depression, Emotional):ti,ab,kw OR (central depression):ti,ab,kw OR (clinical depression):ti,ab,kw | 68694 |
| #19 | (depressive disease):ti,ab,kw OR (depressive disorder):ti,ab,kw OR (depressive episode):ti,ab,kw OR (depressive illness):ti,ab,kw OR (depressive personality disorder):ti,ab,kw | 27707 |
| #20 | (depressive state):ti,ab,kw OR (depressive symptom):ti,ab,kw OR (depressive syndrome):ti,ab,kw OR (mental depression):ti,ab,kw OR (Anxiety):ti,ab,kw | 86673 |
| #21 | (Angst):ti,ab,kw OR (Social Anxiety):ti,ab,kw OR (Anxieties, Social):ti,ab,kw OR (Hypervigilance):ti,ab,kw OR (Nervousness):ti,ab,kw | 12301 |
| #22 | (Anxiousness):ti,ab,kw OR (Stress):ti,ab,kw OR (alarm reaction):ti,ab,kw OR (biologic stress):ti,ab,kw OR (biological stress):ti,ab,kw | 76503 |
| #23 | (organismal stress):ti,ab,kw OR (physiologic stress):ti,ab,kw OR (physiological stresses):ti,ab,kw OR (stress reaction):ti,ab,kw OR (stress resistance):ti,ab,kw | 7763 |
| #24 | (stress response):ti,ab,kw OR (stress situation):ti,ab,kw OR (Substance-use):ti,ab,kw OR (Substance Related Disorder):ti,ab,kw OR (Related Disorders, Substance):ti,ab,kw | 24827 |
| #25 | (Drug Use Disorders):ti,ab,kw OR (Drug Use Disorder):ti,ab,kw OR (Organic Mental Disorders):ti,ab,kw OR (Substance-Induced):ti,ab,kw OR (Abuse, Substance):ti,ab,kw | 46868 |
| #26 | (Substance Abuses):ti,ab,kw OR (Substance Dependence):ti,ab,kw OR (Substance Addiction):ti,ab,kw OR (Chemical Dependence):ti,ab,kw OR (Chemical Dependences):ti,ab,kw | 5167 |
| #27 | (Dependence, Drug):ti,ab,kw OR (Addiction, Drug):ti,ab,kw OR (Prescription Drug Abuse):ti,ab,kw OR (Substance Use):ti,ab,kw OR (Substance Uses):ti,ab,kw | 23110 |
| #28 | (Drug Abuse):ti,ab,kw OR (Habituation, Drug):ti,ab,kw OR (Substance Use Disorders):ti,ab,kw OR (attention-deficit):ti,ab,kw OR (attention deficit and disruptive behaviour disorders):ti,ab,kw | 20307 |
| #29 | (attention deficit disorder):ti,ab,kw OR (attention deficit disorder with hyperactivity):ti,ab,kw OR (ADHD):ti,ab,kw | 7569 |
| #30 | #17 or #18 or #19 or #20 or #21 or #22 or #23 or #24 or #25 or #26 or #27 or #28 | 242967 |
| #31 | #16 and #30 | 1534 |

**3.Embase**

| Search number | Query | Results |
| --- | --- | --- |
| #1 | 'distress syndrome'/exp | 61520 |
| #2 | 'psychological distress'/exp OR 'psychological distress' OR (psychological AND ('distress'/exp OR distress)) OR 'distress, psychological':ab,ti OR 'emotional distress':ab,ti OR 'emotional stress':ab,ti OR 'dystress syndrome':ab,ti OR 'depressive symptoms':ab,ti OR 'depression, emotional':ab,ti OR 'central depression':ab,ti OR 'clinical depression':ab,ti OR 'depressive disease':ab,ti OR 'depressive disorder':ab,ti OR 'depressive episode':ab,ti OR 'depressive illness':ab,ti OR 'depressive personality disorder':ab,ti OR 'depressive state':ab,ti OR 'depressive symptom':ab,ti OR 'depressive syndrome':ab,ti OR 'mental depression':ab,ti OR angst:ab,ti OR 'social anxiety':ab,ti OR 'anxieties, social':ab,ti OR hypervigilance:ab,ti OR nervousness:ab,ti OR anxiousness:ab,ti OR 'alarm reaction':ab,ti OR 'biologic stress':ab,ti OR 'biological stress':ab,ti OR 'organismal stress':ab,ti OR 'physiologic stress':ab,ti OR 'physiological stresses':ab,ti OR 'stress reaction':ab,ti OR 'stress resistance':ab,ti OR 'stress response':ab,ti OR 'stress situation':ab,ti OR 'stress, physiological':ab,ti OR 'substance related disorder':ab,ti OR 'related disorders, substance':ab,ti OR 'drug use disorders':ab,ti OR 'organic mental disorders, substance-induced':ab,ti OR 'abuse, substance':ab,ti OR 'substance abuses':ab,ti OR 'substance dependence':ab,ti OR 'substance addiction':ab,ti OR 'chemical dependence':ab,ti OR 'chemical dependences':ab,ti OR 'dependence, drug':ab,ti OR 'addiction, drug':ab,ti OR 'prescription drug abuse':ab,ti OR 'substance use':ab,ti OR 'substance uses':ab,ti OR 'drug abuse':ab,ti OR 'habituation, drug':ab,ti OR 'substance use disorders':ab,ti OR 'attention deficit':ab,ti OR ('attention deficit':ab,ti AND 'disruptive behaviour disorders':ab,ti) OR 'attention deficit disorder':ab,ti OR 'attention deficit disorder with hyperactivity':ab,ti | 487434 |
| #3 | #1 OR #2 | 487434 |
| #4 | 'internet addiction'/exp | 3764 |
| #5 | 'internet addiction'/exp OR 'internet addiction' OR (('internet'/exp OR internet) AND ('addiction'/exp OR addiction)) OR 'internet gaming disorder':ab,ti OR 'internet use disorder':ab,ti OR 'problematic internet use':ab,ti OR 'sexual preoccupations':ab,ti OR (social:ab,ti AND 'excessive email/text':ab,ti) OR 'internet dependency':ab,ti OR 'computer addiction':ab,ti OR 'addiction disorder, internet':ab,ti OR 'addiction, social media':ab,ti OR 'addictions, social media':ab,ti OR 'addiction, smartphone':ab,ti OR 'addictions, smartphone':ab,ti OR 'disorders, internet gaming':ab,ti OR 'compulsive internet usage':ab,ti OR 'depressive symptom':ab,ti OR 'compulsive internet use':ab,ti OR 'excessive internet usage':ab,ti OR 'excessive internet use':ab,ti OR 'internet dependence':ab,ti OR 'internet over-use':ab,ti OR hypervigilance:ab,ti OR 'internet overusage':ab,ti OR 'internet overuse':ab,ti OR 'online addiction':ab,ti OR 'online media addiction':ab,ti OR 'pathologic internet use':ab,ti OR 'pathological internet usage':ab,ti OR 'pathological internet use':ab,ti OR 'problematic internet usage':ab,ti | 16316 |
| #6 | #4 OR #5 | 16316 |
| #7 | #3 AND #6 | 8200 |

**4.Web of science**

| Search number | Query | Results |
| --- | --- | --- |
| #1 | TI=（Psychological Distress OR Distress, Psychological Emotional Distress OR Emotional Stress OR dystress syndrome OR Depression OR Depressive Symptoms OR Depressive Symptom OR Depression, Emotional OR central depression OR clinical depression OR depressive disease OR depressive disorder OR depressive episode OR depressive illness OR depressive personality disorder OR depressive state OR depressive symptom OR depressive syndrome OR mental depression OR Anxiety OR Angst OR Social Anxiety OR Anxieties, Social OR Hypervigilance OR Nervousness OR Anxiousness Stress OR alarm reaction OR biologic stress OR biological stress OR organismal stress OR physiologic stress OR physiological stresses OR stress reaction OR stress resistance OR stress response OR stress situation OR stress, physiological OR Substance-use OR Substance Related Disorder OR Related Disorders, Substance OR Drug Use Disorders OR Drug Use Disorder OR Organic Mental Disorders OR Substance-Induced OR Abuse, Substance OR Substance Abuses OR Substance Dependence OR Substance Addiction OR Chemical Dependence OR Chemical Dependences OR Dependence, Drug OR Addiction, Drug OR Prescription Drug Abuse OR Substance Use OR Substance Uses OR Drug Abuse OR Habituation, Drug OR Substance Use Disorders OR attention-deficit OR ADHD OR attention deficit and disruptive behaviour disorders OR attention deficit disorder OR attention deficit disorder with hyperactivity）AND AB=（Psychological Distress OR Distress, Psychological Emotional Distress OR Emotional Stress OR dystress syndrome OR Depression OR Depressive Symptoms OR Depressive Symptom OR Depression, Emotional OR central depression OR clinical depression OR depressive disease OR depressive disorder OR depressive episode OR depressive illness OR depressive personality disorder OR depressive state OR depressive symptom OR depressive syndrome OR mental depression OR Anxiety OR Angst OR Social Anxiety OR Anxieties, Social OR Hypervigilance OR Nervousness OR Anxiousness Stress OR alarm reaction OR biologic stress OR biological stress OR organismal stress OR physiologic stress OR physiological stresses OR stress reaction OR stress resistance OR stress response OR stress situation OR stress, physiological OR Substance-use OR Substance Related Disorder OR Related Disorders, Substance OR Drug Use Disorders OR Drug Use Disorder OR Organic Mental Disorders OR Substance-Induced OR Abuse, Substance OR Substance Abuses OR Substance Dependence OR Substance Addiction OR Chemical Dependence OR Chemical Dependences OR Dependence, Drug OR Addiction, Drug OR Prescription Drug Abuse OR Substance Use OR Substance Uses OR Drug Abuse OR Habituation, Drug OR Substance Use Disorders OR attention-deficit OR ADHD OR attention deficit and disruptive behaviour disorders OR attention deficit disorder OR attention deficit disorder with hyperactivity） | 291885 |
| #2 | TI=（Internet addiction disorders OR Internet gaming disorderORInternet use disorder OR excessive internet use OR problematic internet use OR sexual preoccupations OR Social excessive email OR Internet dependency computer addiction OR Addiction Disorder OR Internet Addiction OR Social Media OR Addictions, Social Media OR Addiction, Smartphone OR Addictions, Smartphone OR Disorders, Internet Gaming OR compulsive internet usage OR compulsive internet use OR excessive internet usage OR excessive internet use OR internet dependence OR internet over-use OR internet overusage OR internet overuse OR online addiction OR online media addiction OR pathologic internet use OR pathological internet usage OR pathological internet use  OR problematic internet usage）AND AB=（Internet addiction disorders OR Internet gaming disorderORInternet use disorder OR excessive internet use OR problematic internet use OR sexual preoccupations OR Social excessive email OR Internet dependency computer addiction OR Addiction Disorder OR Internet Addiction OR Social Media OR Addictions, Social Media OR Addiction, Smartphone OR Addictions, Smartphone OR Disorders, Internet Gaming OR compulsive internet usage OR compulsive internet use OR excessive internet usage OR excessive internet use OR internet dependence OR internet over-use OR internet overusage OR internet overuse OR online addiction OR online media addiction OR pathologic internet use OR pathological internet usage OR pathological internet use OR problematic internet usage） | 34502 |
| #3 | #1 AND #2 | 1164 |

Table S2. The characteristics of studies included in this meta-analysis.

| Author | Year | Nation | N | Age | Tools for measuring psychosis and sleep | Tools for measuring Internet addiction |
| --- | --- | --- | --- | --- | --- | --- |
| Awasthi et al | 2020 | Uttarakhand | 221 | ≥ 18 | PSQI | IAT |
| Azhari et al | 2022 | Singapore | 41 | ≤ 18 | DASS-21 | SMD |
| Aziz et al | 2018 | Klang Valley | 199 | ≤ 18 | DASS-21 | IAT |
| Bazrafshan et al | 2019 | Iran | 119 | ≥ 18 | BDI | IAT |
| Bisen et al | 2020 | India | 1600 | ≥ 18 | BDI | IAT |
| Chi et al | 2019 | China | 522 | ≤ 18 | CESD | IAT |
| Deb et al | 2022 | India | 258 | ≥ 18 | DASS-21 | IAT |
| Dib et al | 2021 | Lebanon | 1810 | ≤ 18 | ADRS | IAT |
| Fekih-Romdhane et al | 2022 | Tunis | 114 | ≥ 18 | DASS-21 | IAT |
| Ge et al | 2023 | Arab Emirates | 421 | ≤ 18 | CESD | SAS |
| Geng et al | 2021 | China | 355 | ≥ 18 | DASS-21 | SAS |
| Gundogdu et al | 2022 | Turkey | 244 | ≤ 18 | SCARED/PSQI | PIUS |
| Gupta et al | 2021 | India | 292 | ≥ 18 | PHQ-9/PSQI | IAT |
| Haand et al | 2020 | Afghanistan | 329 | ≥ 18 | CESD | IAT |
| Haddad et al | 2021 | Lebanon | 1103 | ≤ 18 | ADRS | IAT |
| Hsieh et al | 2018 | Taiwan | 500 | ≥ 18 | BDI | IAT |
| Javaeed et al | 2019 | Kashmir | 210 | ≥ 18 | DASS-21 | IAT |
| Jiang et al | 2022 | China | 2688 | ≥ 18 | SDS/PSQI | IAT |
| Karakose et al | 2022 | Turkey | 332 | ≥ 18 | DASS-21 | IAT |
| Kayis et al | 2022 | Turkey | 110 | ≥ 18 | DASS-21 | SAS |
| Kim et al | 2019 | South Korea | 4521 | ≤ 18 | BDI | IAT |
| Kożybska et al | 2022 | Poland | 538 | ≥ 18 | BDI | IAT |
| Kumar et al | 2018 | Indian | 384 | ≥ 18 | BDI | IAT |
| Lavoie et al | 2023 | Canada | 2883 | ≤ 18 | SCARED | IAT |
| Lebni et al | 2020 | Iran | 166 | ≥ 18 | GHQ | IAT |
| Lee et al | 2022 | South Korea | 1155 | ≥ 18 | PHQ-9 | IAT |
| Lei et al | 2020 | Malaysia | 574 | ≥ 18 | DASS-21 | SAS |
| Mamun et al | 2019 | Bangladesh | 405 | ≥ 18 | DASS-21 | IAT |
| Mamun et al | 2020 | Bangladesh | 605 | ≥ 18 | GHQ | IAT |
| Masaeli et al | 2021 | Iran | 298 | ≥ 18 | PSQI | IAT |
| Paudel et al | 2021 | Nepal | 494 | ≥ 18 | BDI | IAT |
| Peterka-Bonetta et al | 2019 | China | 133 | ≥ 18 | BDI | IAT |
| Przepiorka et al | 2019 | Poland | 718 | ≤ 18 | CESD | IAT |
| Saikia et al | 2019 | Kamrup | 416 | ≤ 18 | DASS-21 | IAT |
| Sami et al | 2018 | Israel | 631 | ≤ 18 | PHQ | IAT |
| Sayed et al | 2022 | Egypt | 808 | ≥ 18 | DASS-21 | IAT |
| Stankovic et al | 2021 | Germen | 92 | ≥ 18 | PSQI | IAT |
| Tian et al | 2021 | China | 1200 | ≤ 18 | CESD | IAT |
| Vally et al | 2020 | Arab Emirates | 697 | ≥ 18 | CESD | PIUQ |
| Vally et al | 2019 | China | 706 | ≥ 18 | CESD | PIUQ |
| Wang et al | 2020 | China | 1087 | ≤ 18 | CESD | GPIUS |
| Wang et al | 2021 | China | 1040 | ≥ 18 | PSQI | IAT |
| Yi et al | 2021 | China | 1545 | ≤ 18 | SDS | IAT |
| Yuan et al | 2021 | China | 1809 | ≥ 18 | DASS-21 | IAT |
| Yücens et al | 2018 | Turkish | 392 | ≥ 18 | BDI | IAT |
| Zhang et al | 2021 | China | 734 | ≤ 18 | CESD | IAT |
| Zhao et al | 2023 | China | 7958 | ≤ 18 | SCARED/CESD | IAT |
| Zhao et al | 2022 | China | 904 | ≤ 18 | CESD | YDQ |

Notes: PSQI, Pittsburg Sleep Quality Index; DASS, The Depression Anxiety Stress Scale; BDI, Beck Depression Inventory; CESD, Center for Epidemiologic Studies Depression Scale; ADRS, Aphasic Depression Rating Scale; SCARED, The Screen for Child Anxiety Related Emotional Disorders; PHQ-9, Patient Health Questionnaire-9; SDS, Self-Rating Depression Scale; GHQ, General Health Questionnaire; IAT, Internet Addiction Test; SMD, Social Media Dependency Questionnaire; SAS, Smartphone Addiction Scale; GPIUS, Generalized Pathological Internet Use Scale; YDQ, Young's Diagnostic Questionnaire.

Table S3. The subgroup analyses revealed the summary correlation coefficient between IAD and depression.

| **Subgroup** | **Numbers of studies** | **r** | **95%CI** | **p** | **I^2^** |
| --- | --- | --- | --- | --- | --- |
| **age** | | | | | |
| < 18 | 13 | 0.35 | 0.34-0.36 | 0.000 | 96.5% |
| ≥ 18 | 20 | 0.32 | 0.31-0.34 | 0.000 | 91.2% |
| **Geographic location** | | | | | |
| Arab Emirates | 2 | 0.17 | 0.11-0.22 | 0.000 | 98.0% |
| China | 13 | 0.38 | 0.36-0.39 | 0.000 | 91.8% |
| Poland | 2 | 0.42 | 0.37-0.48 | 0.010 | 85.0% |
| Turkey | 3 | 0.19 | 0.09-0.28 | 0.109 | 61.2% |
| Iran | 3 | 0.12 | 0.01-0.24 | 0.163 | 48.7% |
| South Korea | 2 | 0.44 | 0.24-0.65 | 0.000 | 97.4% |
| Lebanon | 2 | 0.13 | 0.10-0.17 | 0.000 | 98.4% |
| **Questionnaires** | | | | | |
| CESD | 10 | 0.39 | 0.38-0.41 | 0.000 | 94.5% |
| DASS-21 | 15 | 0.30 | 0.27-0.33 | 0.001 | 68.6% |
| BDI | 9 | 0.34 | 0.32-0.37 | 0.378 | 3.0% |
| PHQ | 3 | 0.47 | 0.43-0.51 | 0.000 | 91.7% |
| GHQ | 2 | 0.12 | 0.05-0.19 | 0.314 | 1.4% |
| ADRS | 2 | 0.13 | 0.10-0.17 | 0.000 | 98.4% |
| SDS | 2 | 0.33 | 0.30-0.36 | 0.000 | 94.4% |

Notes: PSQI, Pittsburg Sleep Quality Index; DASS, The Depression Anxiety Stress Scale; BDI, Beck Depression Inventory; CESD, Center for Epidemiologic Studies Depression Scale; ADRS, Aphasic Depression Rating Scale; SCARED, The Screen for Child Anxiety Related Emotional Disorders; PHQ-9, Patient Health Questionnaire-9; SDS, Self-Rating Depression Scale; GHQ, General Health Questionnaire

Table S4. The subgroup analyses revealed the summary correlation coefficient between IAD and anxiety.

| **Subgroup** | **Numbers of studies** | **r** | **95%CI** | **p** | **I^2^** |
| --- | --- | --- | --- | --- | --- |
| **age** | | | | | |
| < 18 | 5 | 0.37 | 0.35-0.38 | 0.000 | 93.7% |
| ≥ 18 | 6 | 0.30 | 0.25-0.35 | 0.000 | 79.8% |
| **Questionnaires** | | | | | |
| DASS-21 | 8 | 0.32 | 0.28-0.36 | 0.000 | 74.1% |
| SCARED | 3 | 0.37 | 0.35-0.38 | 0.000 | 96.8% |

Figure S1 Forest plot of overall SMDs comparing depression scores among Internet addicted groups and non Internet addicted groups.


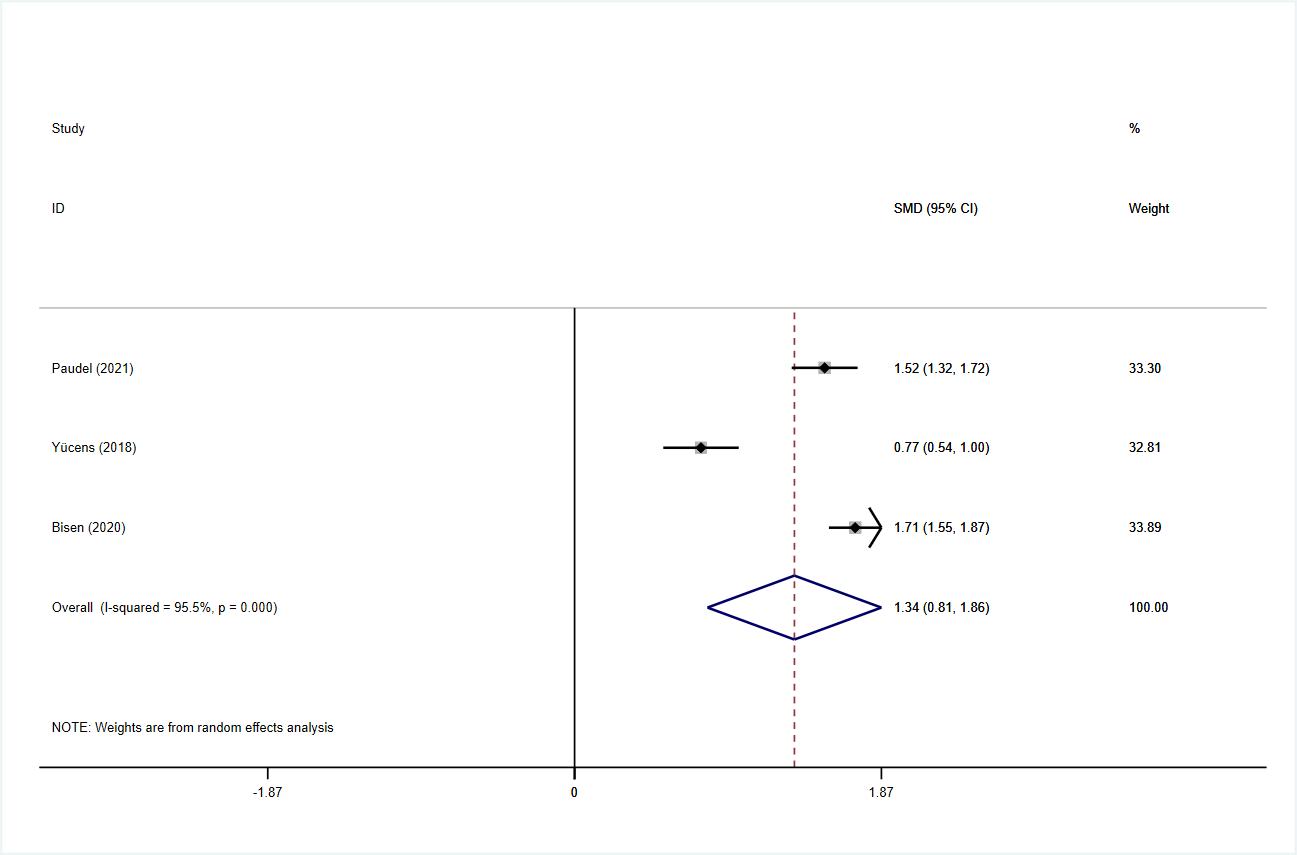


Figure S2 Forest plot of overall ORs for depression among IAD individuals.


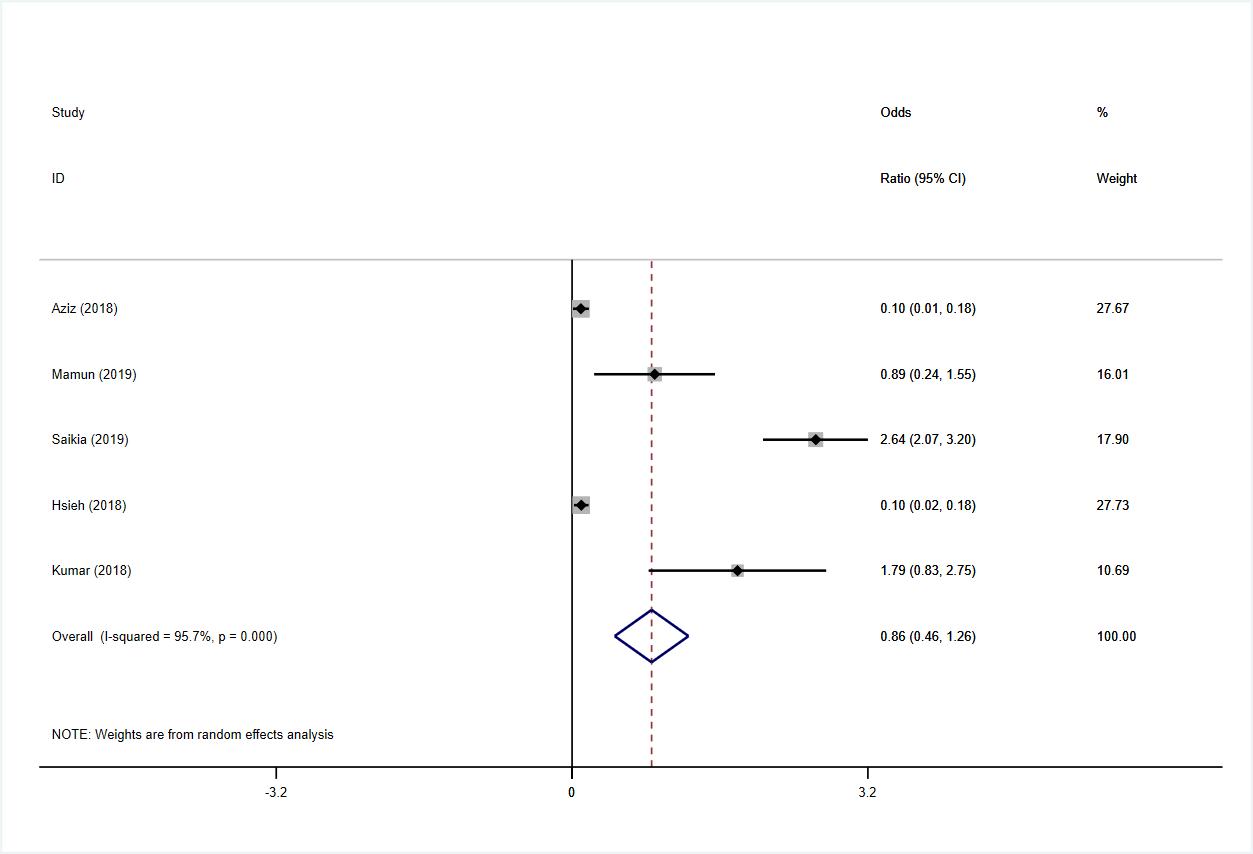


Figure S3 The funnel plots of the association between IAD and depression.


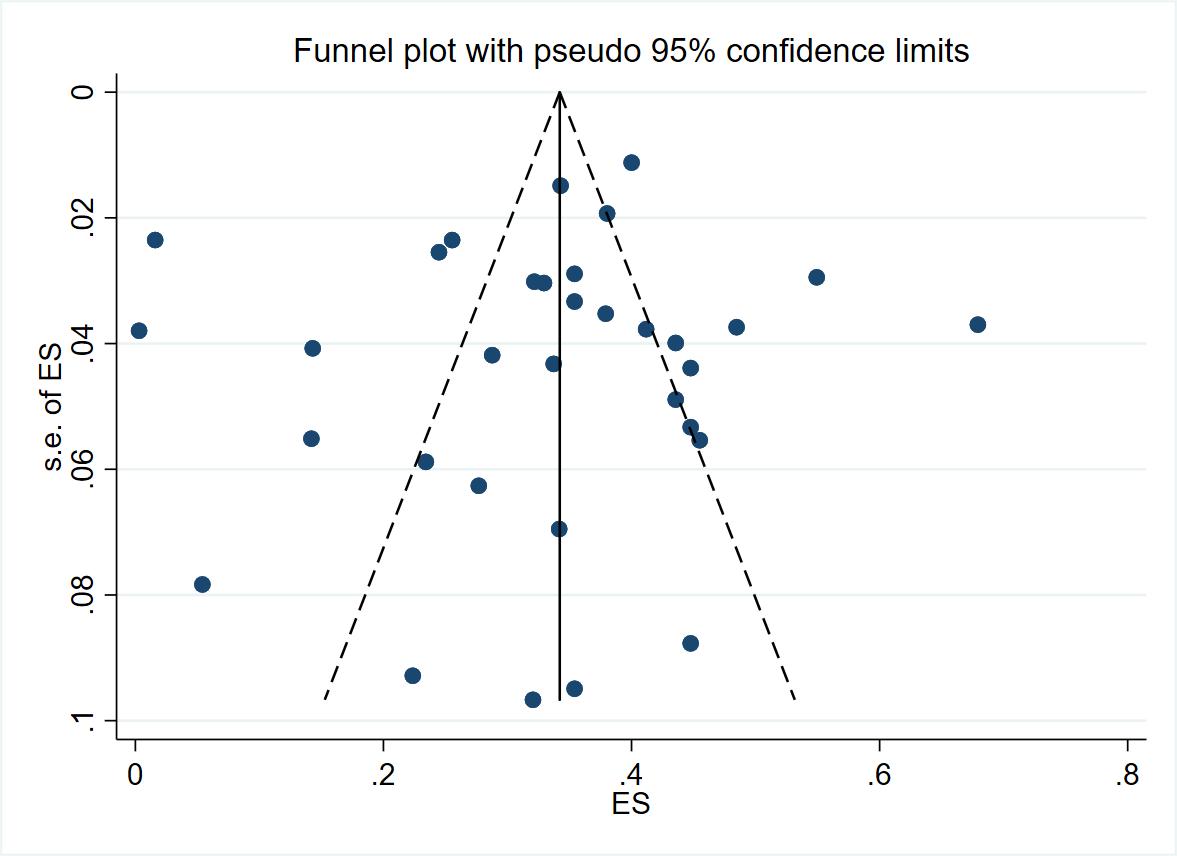


Figure S4 Forest plot of the overall pooled OR of anxiety and Internet addiction.


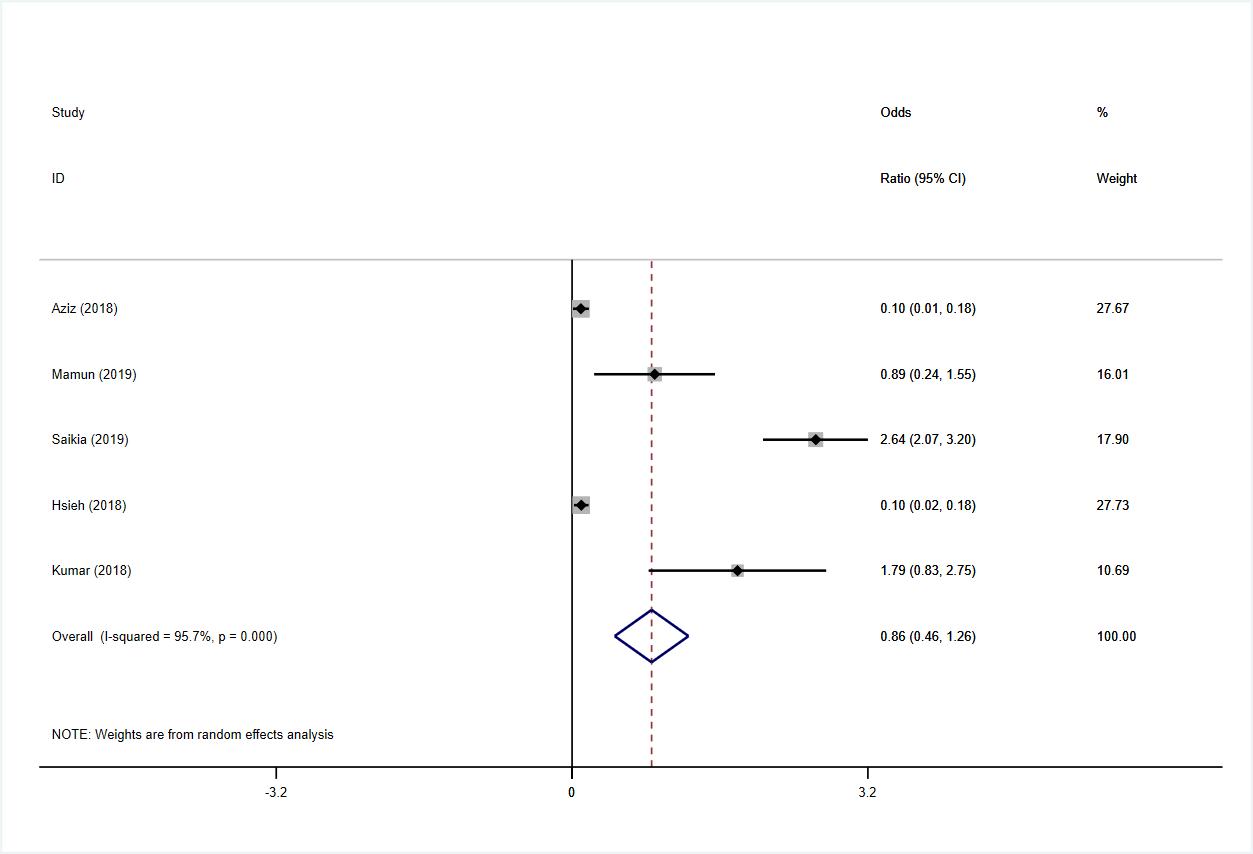


Figure S5 The funnel plots of the association between IAD and anxiety.


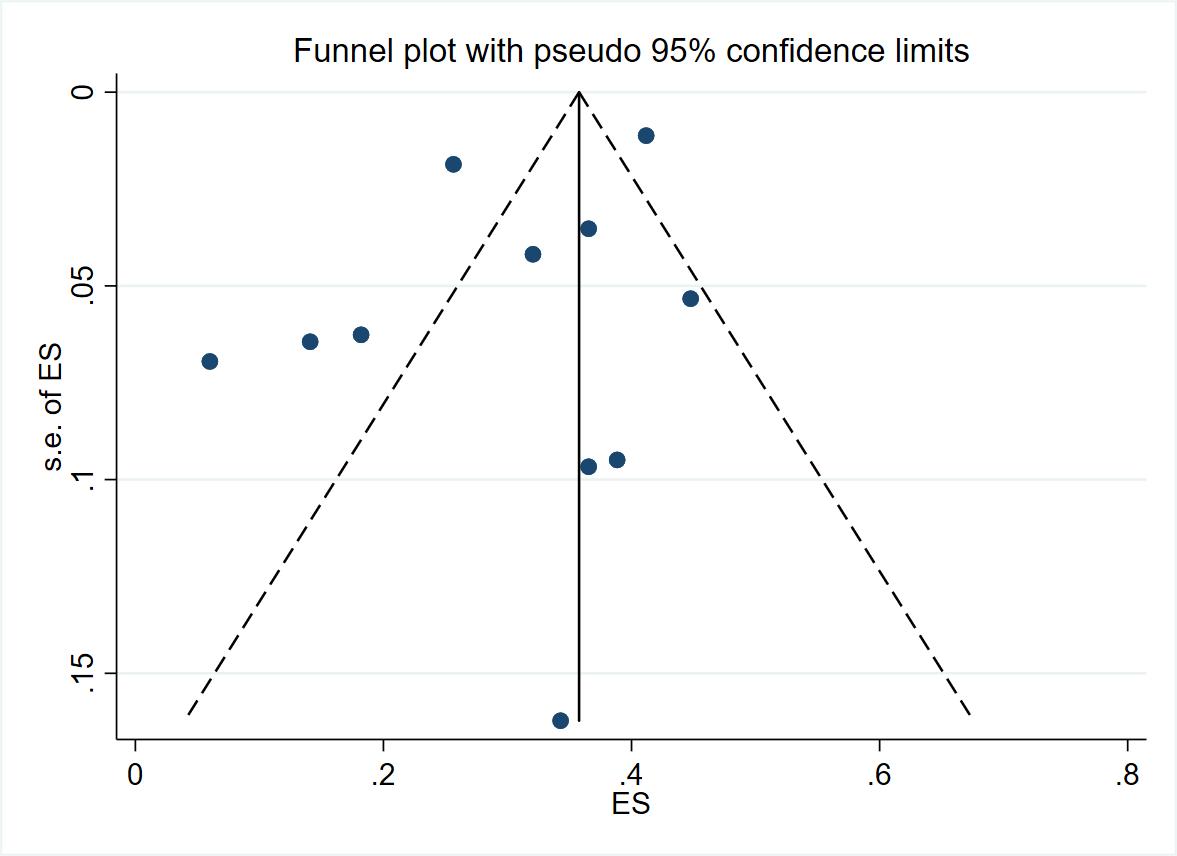


Figure S6 The funnel plots of the association between IAD and sleep quality.


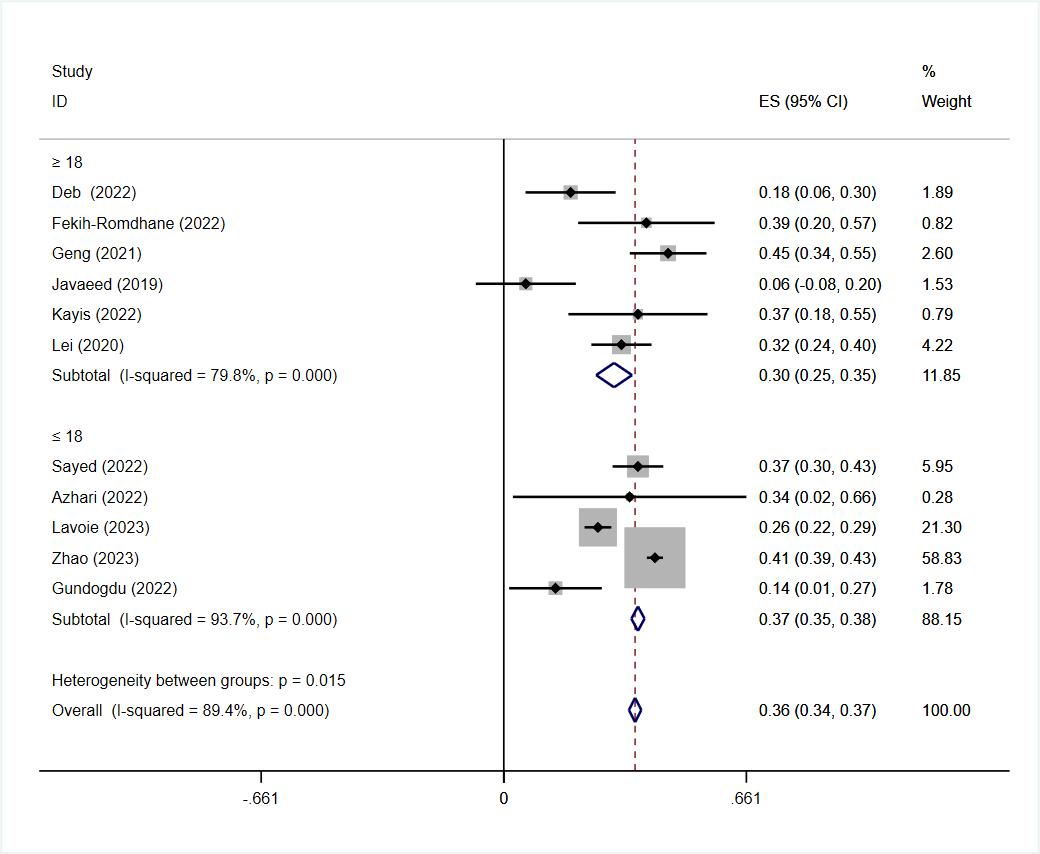

Supplement: Supplementary file 1 [file Table_1.DOCX]
